# Similarity-guided swarm of models: enhancing semi-supervised learning in computational pathology

**DOI:** 10.1038/s41598-025-33281-3

**Published:** 2025-12-30

**Authors:** Zhilong Weng, Alexey Pryalukhin, Wolfgang Hulla, Andrey Bychkov, Junya Fukuoka, Simon Schallenberg, Oliver Buchstab, Frederik Klauschen, Reinhard Büttner, Yuri Tolkach

**Affiliations:** 1 Institute of Pathology, University Hospital Cologne, Kerpener Str. 62, 50937 Cologne, Germany; 2 Institute of Pathology, University Hospital Wiener, Neustadt / Danube Private University, Wiener Neustadt, Austria; 3 Kameda Medical Center, Kamogawa, Japan; 4 Department of Pathology Informatics, Nagasaki University Graduate School of Biomedical Sciences, Nagasaki, Japan; 5 Institute of Pathology, Charité, Berlin, Germany; 6 Institute of Pathology, Ludwig Maximilian University of Munich, Munich, Germany

**Keywords:** Semi-supervised learning, Colorectal cancer, Swarm-of-models, Similarity, Segmentation, Cancer, Computational biology and bioinformatics, Mathematics and computing

## Abstract

High-precision pixel-level annotation has been a major bottleneck in computational pathology due to its time-consuming nature and reliance on expert knowledge. Semi-supervised learning (SSL) provides a promising approach to alleviate this challenge by leveraging large amounts of unlabeled data. However, existing pseudo-labeling-based SSL methods often overlook intrinsic properties, such as inter-case similarities, which are critical for generating accurate pseudo-labels in complex tissue environments. In this study, we propose a Swarm-of-Models (S–o-M) SSL framework that dynamically selects “morphology expert” models (i.e., models specialized in recognizing specific tissue structures) for each unlabeled whole-slide image (WSI) based on similarity, thereby improving the reliability of pseudo-labeling for semantic segmentation tasks. In an evaluation on a large international dataset (multi-class tissue segmentation algorithm for colorectal domain), our approach outperforms traditional supervised and semi-supervised strategies by improving the Dice score by 3.6% for tumor segmentation and 2.1% for tumor/tumor stroma segmentation. Ablation studies performed with different numbers of annotated and unannotated WSIs, as well as training in a monocentric training scenario, further confirm the robustness and superior performance of the proposed S–o-M framework. These findings highlight the value of incorporating case-to-case similarities into SSL strategies to build more effective and general computational pathology models.

**Supplementary Information:**

The online version contains supplementary material available at 10.1038/s41598-025-33281-3.

## Introduction

Pathology is undergoing a profound transformation with the integration of digital technologies, offering clinicians advanced tools for faster and more accurate analysis of medical images^1–4^ . Among these advancements, artificial intelligence (AI) has demonstrated remarkable potential in automating critical tasks such as tumor detection, disease categorization, and the classification of various pathological conditions^5–7^. In oncological pathology, AI-driven tools are increasingly utilized to control the quality of the slides^8^, automate diagnostic workflows^3,4,9–11^, predict patient prognosis^1,12,13^, responses to treatment^14–16^, and, ultimately, paving the way for more personalized and precise healthcare.

In recent years, pathology AI has advanced rapidly. Fully supervised networks, such as Hover-Net^17^ and U-Net^18^ variants, have achieved highly accurate nuclei and tissue segmentation, enabling downstream tasks such as cellular morphology quantification and diagnostic analysis. Weakly supervised learning, particularly multiple-instance learning (MIL), has become the standard approach for training on large-scale whole-slide images (WSIs) with only slide-level labels. Recent developments, including attention-based MIL and hierarchical aggregation models, have improved both model performance and interpretability. Self-supervised learning methods, such as DINO^19^, effectively leverage large unlabeled histology archives. These approaches produce domain-specific feature encoders that often outperform conventional ImageNet pretraining on many pathology tasks. More recently, pathology foundation models, including vision-language architectures and whole-slide transformer networks, have demonstrated strong generalizability and sample efficiency. They support a wide range of applications, including cancer subtyping, tumor microenvironment characterization, and molecular biomarker prediction directly from H&E slides. These advances reflect a shift in computational pathology from labor-intensive, annotation-heavy supervised training toward scalable, label-efficient, and multimodal representation learning.

Despite these promising developments, the deployment of AI in computational pathology encounters significant challenges. Particularly the need for large, manually annotated datasets to train robust diagnostic algorithms with state-of-the-art, pixel-wise precision^20^. Unlike datasets in other fields, annotating whole-slide pathology images (WSIs) requires highly specialized expertise and is a very laborious task. This limitation presents a major barrier to the advancement of AI in pathology.

To address this challenge, semi-supervised learning (SSL) methods are being increasingly adopted in development of AI algorithms for medical imaging^21,22^. Approaches such as pseudo-labeling and consistency regularization can facilitate the development of effective models using limited labeled data supplemented with large unlabeled datasets^23,24^. Among these, pseudo-label-based methods have shown notable success across various domains^25^. However, the efficacy of these methods is strongly dependent on the quality of the pseudo-labels, which directly affects model performance^26^. For instance, ERSR^27^ improves pseudo-label accuracy and reliability in medical image segmentation by introducing constraints and optimization steps, such as an ellipse-based regularization. Similarly, IP-ACPS^28^ leverages iterative pseudo-labeling with adaptive copy-paste supervision to enhance small and sparse tumor segmentation, while the uncertainty-based feature aggregation model^29^ exploits inter- and intra-slide uncertainty to refine pseudo-label quality and guide feature aggregation for improved histopathology segmentation.

In this study, our *main contribution* is to propose a novel SSL approach specifically designed for digital pathology and semantic segmentation tasks. It implements a “swarm” (pool) of models, each being a “morphology expert” in certain tumor morphology. For each new unlabeled WSI, one (most suitable) model is being selected for pseudo-labeling, based on developed similarity assessment principle involving state-of-the-art foundational pathology feature encoder. This allows for generation of high-quality pseudo-labels for training tissue classes with limited amount of labeled data, using this sparse data in the most flexible and fine-granular way. The method has been extensively validated using independent datasets from multiple sites to ensure its reliability. Furthermore, to foster reproducibility and broader application, we have open-sourced the relevant code, enabling researchers to verify and adapt the method for a wide range of use cases.

## Materials and methods

### Training datasets

For algorithm development, we use a dataset of 245 WSIs (hematoxylin and eosin-stained, H&E) from colorectal cancer cohort of The Cancer Genome Atlas (TCGA) that were manually annotated by pathology experts with high-quality, pixel-level precision^10^. Additionally, 30 annotated WSIs and 200 non-annotated WSIs from resection specimen cases were collected from University Hospital Cologne (UKK). The TCGA dataset includes samples from a wide range of institutions (n = 36) and laboratory procedures, while UKK is a single institution dataset. Most WSIs in the training dataset were scanned at 40 × magnification, with a micrometers-per-pixel (MPP) resolution of approximately 0.25, using Leica scanner family (TCGA – Aperio AT2, UKK – GT450). For training the main models—including the supervised learning model, the swarm of models, and the semi-supervised learning model—patches were extracted at 10 × magnification with a size of 512 × 512 pixels. For annotated WSIs, patches were extracted with an overlap of 128 pixels to ensure sufficient coverage of each case. For non-annotated WSIs, patches were extracted without overlap to reduce computational and training time due to the large dataset size.

In total, 245 WSIs from the TCGA cohort contained pixel-level annotations and served as the common source for both annotated and non-annotated data in this study. For each experiment, subsets of these WSIs were randomly selected to serve as annotated WSIs (n = 5, 10, 15) and non-annotated WSIs (n = 20, 50, 100, 200), according to the experimental configuration. Additionally, 30 annotated WSIs and 200 non-annotated WSIs from the UKK cohort were included for ablation studies. Details of each configuration are provided in the Results section.

### Independent test datasets

Several datasets were used from five independent pathology departments: Wiener Neustadt, Austria (WNS); Ludwig Maximilian University of Munich, Germany (LMU); Universitätsmedizin Charite Berlin, Germany (CHA); modified colorectal adenocarcinoma gland (CRAG) dataset^10,30^, and Kameda Medical Center, Kamogawa, Japan (KAM). These slides were digitized using different scanners (WNS: Hamamatsu NanoZoomer S360, LMU: Leica Aperio GT450, CHA: Leica Aperio GT450, CRAG: Omnyx VL120, KAM: Philips UFS) at varying magnifications (MPP 0.25–0.50), and extensively manually annotated by pathology experts with pixel-level precision. Patches were extracted in the same manner as for the training datasets, but with no overlap.

### Annotation principle

All annotations were created in QuPath software^31^ using “dense” principle. Shortly, the rectangular regions representative of tumor case were selected by pathologists and pixel-dense annotations were created where possible. Eleven histological classes were annotated in H&E-stained whole-slide images (WSIs): tumor tissue, tumor stroma, benign mucosa, submucosal tissue (including large vessels), smooth muscle tissue (muscularis propria and muscularis mucosae), adventitial tissue (including large vessels), blood vessels with muscular walls, lymphoid regions (including lymphoid aggregates and lymph nodes), ulceration and necrotic debris, acellular mucin lakes, bleeding areas, and slide background.

### Implementation of new method and algorithm training

**Fig. 1:**
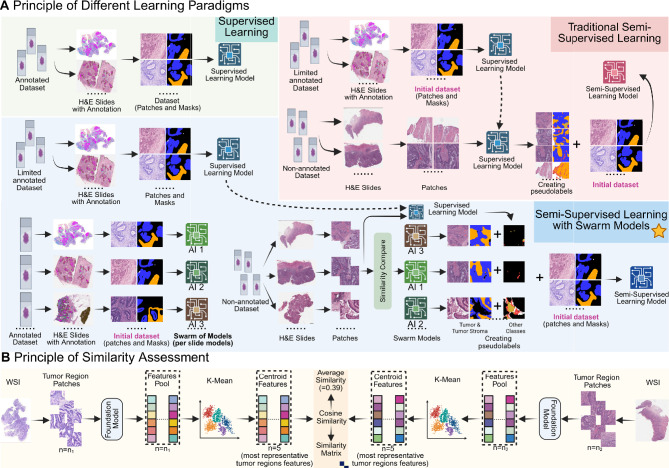
Concept of the S–o-M semi-supervised learning approach and its place among different learning paradigms. (**A**) Different algorithm training paradigms and concept of the suggested method. Three different learning/training paradigms for semantic segmentation problem in histopathological image analysis are presented: supervised learning (green), traditional pseudo-label-based semi-supervised learning (SSL) (red), and pseudo-label-based SSL with S–o-M(blue). i.) In the supervised learning approach, digitized whole slide images (WSIs) are annotated manually by experts with pixel-wise precision. This is an extremely laborious process and the reason why only few high-quality annotated datasets are available. These annotated WSIs are divided into small patches and corresponding class masks, forming a labeled dataset to train a supervised learning model. ii.) In traditional SSL, a limited number of WSIs with pixel-wise annotations are processed in the same manner to train an initial supervised learning model. Additionally, a large number of WSIs without any annotations are divided into small patches. The trained supervised model is then used to generate pseudo-labels for these unlabeled patches, and the combination of labeled and pseudo-labeled patches is used to train a semi-supervised learning model. The bottleneck for traditional SSL is quality of pseudo-labels which is often suboptimal. iii.) The suggested SSL approach with S–o-M method builds upon traditional SSL by introducing a model pool. A limited number of annotated WSIs are used to train multiple supervised learning models, each corresponding to a specific annotated case. For each unlabeled WSI, to generate pseudo-labels, a model will be selected from the pool that was trained using an annotated case with highest similarity to this non-annotated WSI. These should increase the quality of generated pseudo-labels and result in higher accuracies. (**B**) Principle of Similarity Assessment. Benign classes among different classes have very high levels of similarity that results in high accuracies even when using only few annotated cases. Tumor regions represent the major problems due to high inter-case and intra-case heterogeneity of tumor tissue (consisting of two most important classes – tumor itself and tumor stroma). We use tumor regions to measure similarity between cases. All tumor region patches are first extracted from WSIs and processed using a pathology foundation model to generate feature representations. The K-means algorithm with n = 5 is then used to select the five most representative tumor region clusters. The feature vectors from patches closest to the centroid of each cluster are used for similarity comparisons. For a new case, the same process is applied to obtain its representative tumor region features, and cosine similarity is used to systematic comparison of 5 vectors of first and second case, resulting in 25 comparisons and one average similarity value. This similarity assessment determines the most relevant model from the model pool for pseudo-label generation. The selected model generates pseudo-labels for Tumor and Tumor Stroma, which are combined with other classes and cleaned of artifacts to form the final pseudo-labels for semi-supervised training. Created in https://BioRender.com.

We investigate three training strategies for semantic segmentation in WSIs: supervised learning, traditional pseudo-label-based SSL, and a proposed, SSL with S–o-M approach. We use the traditional pseudo-label approach as the primary baseline, as it is widely adopted in pathology and medical image analysis and provides a simple, reproducible, and fair reference for evaluating the proposed S–o-M approach. Details on technical implementation are provided in Fig. 1A. The supervised model is trained using only a small set of available annotated WSIs. The traditional semi-supervised model uses pseudo-labels generated from this initial supervised model to expand the training set with information from unlabeled subset. In the S–o-M approach, multiple models are trained on individual annotated WSIs (one model = one slide) becoming experts in certain morphologies, each model is initialized with the same publicly available encoder checkpoint (based on ImageNet) and trained using 90% of the patches from the corresponding case, with the remaining 10% used for validation to select the best-performing model. Pseudo-labels for tumor and tumor stroma tissue classes (most important and challenging classes with substantial intra- und intertumoral heterogeneity) are generated using morphology expert model with highest levels of tumor tissue similarity between target and training WSIs. Other tissue classes (which are very similar among cases), such as benign mucosa, submucosa, muscle tissue, adventitial tissue, necrotic areas, and mucin are pseudo-labeled using the supervised model. For each non-annotated WSI, the final pseudo-label mask is generated by fusing the predictions from the expert and supervised models at the patch level. In cases of overlapping predictions at patch boundaries, tumor and tumor stroma predictions from the expert model take precedence over other classes. No confidence threshold is applied to any predictions, in order to ensure fair comparison across methods; applying class-specific thresholds could introduce biases in evaluation metrics and confound the assessment of model performance. This design ensures that observed performance differences reflect the effectiveness of the S–o-M approach itself rather than arbitrary threshold choices. Artifacts in the images were detected and removed using the GrandQC tool^8^ before training the semi-supervised model. This strategy aims to significantly improve pseudo-label quality (The detailed training workflow of the S–o-M framework is summarized in Suppl. Figure 1).

All the algorithms used in this study are pixel-wise semantic segmentation models. The final architecture selected for all models consists of EfficientNetB0 as the encoder and UNet +  + as the decoder, with cross-entropy (CE) loss weighted for different classes to address class imbalance, the Adam optimizer, and a StepLR scheduler with a step size of 10. This configuration was previously identified as the most effective for this task in our internal benchmarking experiments. All models were trained for 36 epochs without early stopping, and the checkpoint achieving the best validation performance was used for evaluation. No additional regularization techniques (e.g., Dropout, L2 weight decay) were applied, as all models exhibited stable convergence without overfitting.

Although UNet +  + was used in this study, the S–o-M framework is architecture-agnostic and can be combined with more advanced segmentation backbones (e.g., PIF-Net^32^, its successor the Local and Long-range Progressive Fusion Network^33^, or DFPNet^34^) to better capture multi-scale and boundary-ambiguous structures. Furthermore, S–o-M can be augmented with complementary modules, such as domain-adaptive feature alignment, Bayesian uncertainty-aware collaborative learning, or registration-based pseudo-label propagation, to further enhance cross-site robustness and pseudo-label quality (see Related Work).

### Principle of similarity assessment

The principle of similarity assessment is shown in Fig. 1B. To estimate similarity, we implement the following approach inspired by expert pathology domain knowledge. Tumor region in each WSI is tessellated into patches (224 × 224 with 10 × magnification), the image size was adjusted to correspond to the input specifications of the foundational image encoder employed in the subsequent analysis. For WSIs of the annotated subset, the patches were selected based on manual annotations, whereas for non-annotated WSIs (unlabeled data subset), patches were selected using a specially trained tumor segmentation model, which was trained on a limited amount of labeled data using image patches of size 224 × 224 pixels and is capable of segmenting tumor, non-tumor, and background regions. A threshold of 60% to tumor patch content was applied, meaning that only patches containing more than 60% tumor area were retained for downstream similarity assessment. This value was chosen as a trade-off between tumor purity and data sufficiency. Lower thresholds (e.g., 30%) tend to include a larger proportion of background and stromal tissue, introducing noise in the feature space, whereas higher thresholds (e.g., 90%) substantially reduce the available patch pool, which may lead to insufficient representation of intratumoral heterogeneity. Empirically, the 60% cutoff maintained both adequate patch quantity (> 5,000 per slide) and high tumor specificity. Block-level feature embeddings were then computed using the foundational encoder UNI^35^, which is a state-of-the-art, general-purpose foundational self-supervised encoder built upon ViT-Large for pathology pre-trained on large amounts of histopathological data. Image patches were extracted at 10 × magnification for feature generation. The pre-trained weights were used without any fine-tuning, as UNI has been extensively trained to capture transferable morphological representations across diverse tissue types. Two foundational models were considered for initial selection, including another state-of-the-art Prov-Gigapath model^36^. The results of the selection are provided in Suppl. Figure 2. Direct comparison of two models (slide review by pathology experts and distribution metrics in the generated heatmaps) showed substantially more fine-granular capabilities of similarity assessment using UNI. Feature vectors from each tumor were clustered using K-means clustering and cluster n = 5 to identify the five most representative regions within single tumor. The choice of n = 5 was initially guided by pathology expert review as optimally capturing intratumoral heterogeneity in colorectal adenocarcinoma, and further supported by a quantitative sensitivity analysis (n = 3,5,7) showing that n = 5 yields the highest segmentation performance (see Suppl. Figure 3) and Suppl. Figure 4 illustrates the similarities between non-annotated and annotated cases with n = 3 and 5. The feature vectors of patches closest to the centroids (n = 5) were selected for similarity assessment. The similarity between WSIs was calculated as the average pairwise cosine similarity among these representative features (i.e., for two tumors number of comparisons n = 25 for 5 vectors from each side resulting in one average value). Average similarity values were used to detect most similar WSI in course of S–o-M SSL training. The results of two additional similarity metrics, L1 distance and L2 distance, are provided in Suppl. Figures 5 and 6. A direct comparison of all three similarity metrics, based on slide-level similarity scores reviewed by pathology experts, shows that using cosine similarity for similarity evaluation more effectively reflects the differences between slides.

### Ethical aspects

All study procedures were conducted in accordance with the Declaration of Helsinki. This study was approved by the ethical committees of the University of Cologne and Charité Universitätsmedizin (22–1233, Project FED-PATH; joint Cologne/Charité 20–1583), the Ethical Committee of Lower Austria (GS1-EK-4/694–2021) and Kameda Hospital (22–094). The requirement for patient consent was waived as only anonymized, retrospective materials were used.

## Results

### Principle of swarm-of-models (S–o-M) semi-supervised learning and study design

We propose a principally new SSL approach (principle compared to traditional supervised and semi-supervised learning is outlined in Fig. 1A; for details see Methods) that operates with a pool of “morphology expert” models trained using single annotated cases. This approach is especially suitable for low-resource annotation training scenario and distributed trainings (e.g., federated learning) where each annotated slide is used in a maximally effective way. We apply it to the use case of multi-class tissue semantic segmentation in colorectal cancer (tissue classes n = 11 including background; details see Methods). Among the 11 classes, we focus our analysis on tumor tissue and tumor stroma. These two classes are the most clinically important (for downstream analysis). The benign classes show high levels of similarity among patient cases and their representations are quickly learnable using only few examples. On contrary tumor/tumor stroma show significant heterogeneity within individual tumors and among patient cases that warrants multiple samples accurately annotated by pathologists – highly laborious task. At that, proper segmentation of tumor and tumor stroma has critical implications for diagnosis and prognosis.

The framework includes a similarity assessment module (Fig. 1B; for details see Methods) crafted using expert pathology domain knowledge. This extracts representative features from tumor regions and computes cosine similarity between WSIs using 5 feature vectors for each tumor and 25 pairwise comparisons to determine similarity between two cases. This similarity information guides the selection of an “morphology expert” model from a S–o-M, enabling case-specific pseudo-label generation. By tailoring the pseudo-labeling process to each slide, the method enhances label quality without requiring additional data sharing (see Methods). We initially evaluate two state-of-the-art foundation models for pathology (Prov-Gigapath and UNI) and different methods of similarity measurements (cosine, L1, L2 distances) selecting UNI and cosine similarity as providing similarity assessment at higher depth (as estimated by expert pathologists).

**Fig. 2:**
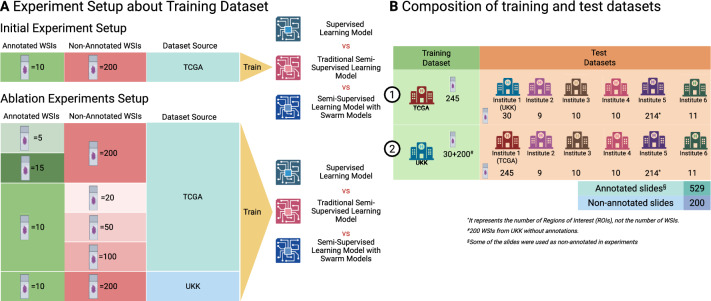
Study design: proof-of-concept and ablation experiment setup. (**A**) In the initial experimental setup, we select the number of annotated and non-annotated slides representing a typical real world use case: 10 annotated WSIs and 200 non-annotated WSIs. The colorectal cohort of the The Cancer Genome Atlas (TCGA) dataset is used for initial experiments as one of the most representative datasets. In initial setup and in ablation experiments, we compare three training approaches—Supervised Learning, traditional semi-supervised learning (SSL), and our SSL approach with S–o-M. During ablation experiments, we study the effects of all relevant hyperparameters on the segmentation accuracy of resulting models (number of annotated slides, number of non-annotated slides, and dataset used for training). (**B**) Cross-Domain Dataset Configuration for Training and Testing. This figure summarizes data contributions from TCGA, UKK, CRAG, and four external institutions. For each source, the number of whole slide images (WSIs) and regions of interest (ROIs) is presented, along with the breakdown of annotated and non-annotated WSIs. This overview highlights the composition of training and test datasets in both initial and ablation experiment setups. Created in https://BioRender.com.

In the following sections, we first demonstrate the proof-of-concept for our approach using a standard experimental setup and then conduct extensive ablation studies to analyze the effects of key hyperparameters. To assess the robustness of our approach, each experimental training setup was repeated three times with independent/randomized subsets of annotated WSIs. Five additional independent test datasets from different laboratories were included (Fig. 2B).

Per-dataset Dice scores for all experiments are provided in Suppl. Table 1.

### Proof-of-concept: Implementation and validation of S–o-M approach using initial experimental setup

**Fig. 3:**
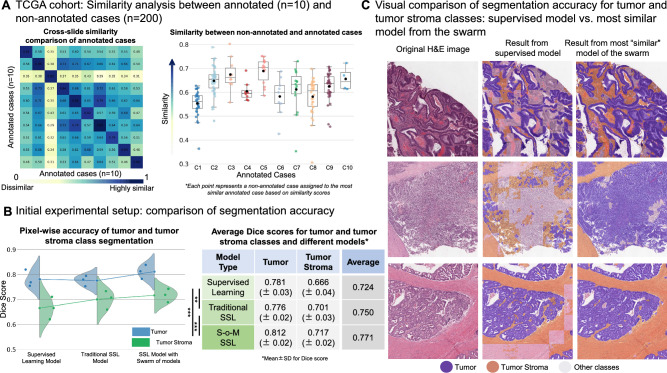
Initial experiments: whole-slide image (WSI) similarity analysis, comparison of segmentation accuracy for different approaches, and visual evaluation of segmentation accuracy (**A**) Similarity analysis between annotated (n = 10) and non-annotated WSIs (n = 200). Slide cohort: TCGA colorectal cancer cohort. The heatmap on the left side depicts the cross-slide case-level similarity values between annotated cases, where darker colors indicate higher similarity. There is a broad range of similarities among unrelated cases (0.29–0.79). The values of similarities for the same cases are higher than for unrelated cases but not equal 1.0 as we take five most representative regions. These values, therefore, outline intra-case intratumoral heterogeneity. The box plot on the right side illustrates the distribution of similarities for a larger cohort of non-annotated cases (n = 200). The non-annotated cases are grouped to the most similar case among annotated cases and the points are similarity values measured against these most similar cases. (**B**) Comparison of segmentation accuracy for different training approaches using initial study setup (TCGA dataset, annotated slide n = 10, non-annotated slide n = 200; details see Fig. 2). Three different approaches (supervised learning, traditional SSL, and SSL using S–o-M) are compared concerning pixel-wise segmentation accuracy (measured by Dice Score). We concentrate on tumor and tumor stroma classes as two bottleneck classes for multi-class tissue segmentation problem (explanation in Fig. 1 and Methods). The accompanying table (right side) provides the average Dice score with standard deviation of 3 independent experiments for each approach, statistical significance of the combined tumor and tumor stroma scores was assessed using a paired t-test (*** p < 0.001, ** p < 0.01). Conclusion: For both tumor and tumor stroma classes, new SSL S–o-M approach provides better segmentation accuracies. (**C**) Visual comparison of segmentation accuracy/generated pseudo-labels in non-annotated slides for supervised model (as part of traditional SSL approach) and for most “similar” model (trained on the most similar annotated case). The review of the images by pathology experts shows higher quality of segmentation/pseudo-labels for S–o-M approach.

To establish the viability of our approach, we adopted a practical experimental setup inspired by the real-world conditions: using only 10 annotated WSIs and 200 non-annotated WSIs/cases from a widely used open-source WSI archive (colorectal cancer cohort of The Cancer Genome Atlas/TCGA). This reflects the typical annotation capacity with limited engagement of pathologists in real-world computational pathology projects, given the high labor and expertise required for manual labeling (up to 5–8 h/case with regional annotation approach^20^). The similarity matrix among the 10 annotated WSIs (first of three independent experiments; for other replicates see Suppl. Figure 7) is shown in Fig. 3A, with cosine similarity scores ranging from 0.29 to 0.89, indicating substantial heterogeneity even within a small annotated slide set and validating the capacity of our similarity principle to detect different morphologies. Figure 3A (right panel) shows the closest annotated match for each of the 200 unannotated WSIs, indicating that inter-slide similarity is a consistent and exploitable property, facilitating targeted pseudo-label generation.

Segmentation accuracy metrics from three models across three independent trials and five independent test datasets are provided in Fig. 3B. Dice scores for tumor and tumor stroma tissue classes were 0.781 and 0.666, 0.776 and 0.701, and 0.812 and 0.717 for supervised approach, traditional semi-supervised approach and our S–o-M, with S–o-M significantly outperforming competitors (paired t-test comparing traditional SSL and S–o-M on combined tumor and tumor stroma scores, p < 0.001, full details, incl. performance on other tissue classes see Suppl. Figure 8, the visual performance of the tumor segmentation models see Suppl. Figure 9, and the segmentation performance of the three models for tumor and tumor stroma across various external datasets see Suppl. Figure 10). Complete performance metrics, including Sensitivity (Recall) and Positive Predictive Value (PPV / Precision) for the main classes (Tumor and Tumor Stroma), are provided in the Suppl. Figure 11. To qualitatively assess pseudo-label accuracy, we randomly selected WSIs from TCGA and extracted representative regions of interest (ROIs), that were visually evaluated by expert pathologists confirming that the S–o-M approach produces noticeably more accurate delineations of tumor and stroma regions (Fig. 3C).

Additionally, we compared with Mean Teacher, which is commonly used in computer vision and increasingly in medical imaging (see Suppl. Figure 12A), for the main tissue types (tumor and tumor stroma), S–o-M achieves performance comparable to the Mean Teacher baseline overall, while outperforming it in 4 of 6 external cohorts. Performance decreases in the remaining two cohorts (one of them – non-standardized CRAG cohort with small regions of interest lacking exact information about resolution), which we traced to low morphological similarity between their cases and the annotated reference set used for model selection (see Suppl. Figure 12B, 12C). Specifically, Institute 5 contains ROI-based images with few tumor patches, limiting reliable similarity estimation, while Institute 3 shows mid-range similarity scores (~ 0.5), consistent with lower segmentation accuracy.

In summary, under realistic conditions, our framework effectively exploits inter-slide similarity within large unlabeled datasets to improve pseudo-labeling and segmentation, providing a strong proof of concept for scalable SSL in computational pathology.

### Ablation experiments

Our proposed similarity-guided framework involves several critical hyperparameters that influence performance: the number of annotated WSIs, the number of non-annotated WSIs, and the dataset composition. While our primary experiments used the TCGA cohort, which is highly heterogeneous and includes samples from 36 different medical centers, a typical clinical setting may rely on data from a single institution, raising concerns about generalizability. In the following sections, we present ablation experiments to systematically evaluate the impact of each of these factors on model performance.

#### Impact of training dataset (single center dataset)

To assess the generalizability of our method under realistic constraints, we performed an ablation study using data exclusively from a single institution that reproduces the realistic scenario when only slides from their own institution are being used for training (TCGA is a multicentric dataset). We use a monocentric UKK dataset for training while keeping the training procedures and evaluation metrics consistent with the initial setup.

**Fig. 4:**
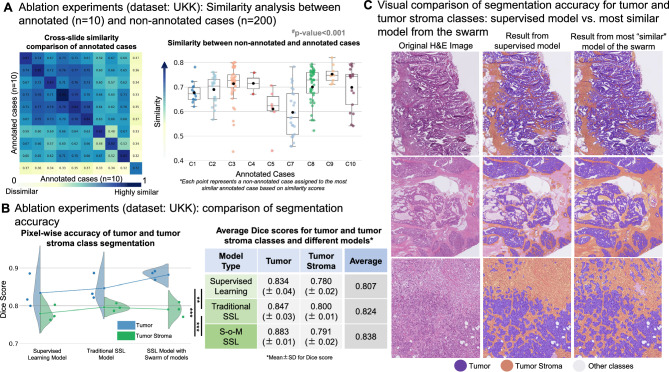
Ablation experiments (dataset): whole-slide image (WSI) similarity analysis, comparison of segmentation accuracy for different approaches, and visual evaluation of segmentation accuracy (**A**) Similarity analysis between annotated (n = 10) and non-annotated WSIs (n = 200). During this ablation experiment we use a different, single-center cohort of cases (UKK colorectal cancer cohort). The heatmap on the left side depicts the cross-slide case-level similarity values between annotated cases, where darker colors indicate higher similarity. The unrelated cases show higher similarity values compared to multi-centric TCGA cohort (Fig. 3A) due to similar cutting and staining quality. This difference is statistically significant based on a two-sample t-test (t = 10.38, p = 1.13 × 10⁻^22^). The box plot on the right side illustrates the distribution of similarities for a larger cohort of non-annotated cases (n = 200). The non-annotated cases are grouped to the most similar case among annotated cases and the points are similarity values measured against these most similar cases. (**B**) Comparison of segmentation accuracy for different training approaches (UKK dataset, annotated slide n = 10, non-annotated slide n = 200). Three different approaches (supervised learning, traditional SSL, and SSL using S–o-M) are compared concerning pixel-wise segmentation accuracy (measured by Dice Score). We concentrate on tumor and tumor stroma classes as two bottleneck classes for multi-class tissue segmentation problem (explanation in Fig. 1 and Methods). The accompanying table (right side) provides the average Dice score with standard deviation of 3 independent experiments for each approach. Conclusion: For tumor class, new SSL S–o-M approach provides significantly better segmentation accuracies, statistical significance of the combined tumor and tumor stroma scores was assessed using a paired t-test (*** p < 0.001, ** p < 0.01); segmentation for tumor stroma class is better compared to supervised model and comparable to traditional SSL (with higher average Dice scores for S–o-M approach). (**C**) Visual comparison of segmentation accuracy/generated pseudo-labels in non-annotated slides for supervised model (as part of traditional SSL approach) and for most “similar” model (trained on the most similar annotated case). The review of the images by pathology experts shows higher quality of segmentation/pseudo-labels for S–o-M approach.

Similarity analysis among annotated and non-annotated WSIs (Fig. 4A) follows the distribution in initial experiment, highlighting substantial variability even within a single source and reinforcing the utility of developed similarity assessment method (for results in two other independent experiments see Suppl. Figure 13). We validated performance on a test set of 285 WSIs and 214 densely annotated ROIs from TCGA, CRAG and four external institutions (for full details incl. performance on other tissue classes see Suppl. Figure 14, the visual performance of the tumor segmentation models see Suppl. Figure 15, and the segmentation performance of the three models for tumor and tumor stroma across various external datasets see Suppl. Figure 16). Like in initial setup, S–o-M model significantly outperformed supervised model and traditional SSL for tumor tissue segmentation and showed similar results for stroma segmentation (Fig. 4B; Dice score for supervised, traditional SSL, and S–o-M 0.834 and 0.780, 0.847 and 0.800, and 0.883 and 0.791 for tumor and tumor stroma classes, paired t-test comparing traditional SSL and S–o-M on combined tumor and tumor stroma scores, p < 0.001). Complete performance metrics, including Sensitivity (Recall) and Positive Predictive Value (PPV / Precision) for the main classes (Tumor and Tumor Stroma), are provided in the Suppl. Figure 11. The S–o-M approach showed improved delineation of tumor and tumor stroma regions in a visual analysis by expert pathologists, validating the effectiveness of suggested approach (Fig. 4C).

#### Impact of number of annotated WSIs

To assess how the number of annotated WSIs affects method’s performance, we conducted experiments with varying quantities of annotated WSIs in a realistic range for a typical computational pathology project (n = 5, 10, and 15), while keeping all other hyperparameters as in initial setup (including non-annotated WSIs n = 200 from TCGA dataset). Evaluation was performed using five independent test datasets like in the initial setup.

**Fig. 5:**
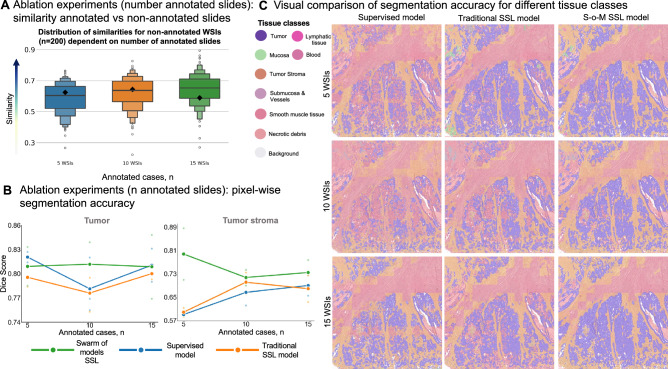
Ablation experiments (number of annotated cases): whole-slide image (WSI) similarity analysis, comparison of segmentation accuracy for different approaches, and visual evaluation of segmentation accuracy. (**A**) Distribution of case-level similarity values for non-annotated WSIs (n = 200; against annotated slides) dependent on the number of used annotated WSIs. During this ablation experiment we evaluate the impact of the number of annotated WSIs included in training (TCGA colorectal cancer cohort). Conclusion: higher number of annotated images included results in higher similarity values for non-annotated slides. (**B**) Comparison of segmentation accuracy for different training approaches (TCGA dataset, annotated slide n = 5, 10 or 15; non-annotated slide n = 200). Three different approaches (supervised learning, traditional SSL, and SSL using S–o-M) are compared concerning pixel-wise segmentation accuracy (measured by Dice Score). Conclusions: new SSL S–o-M approach provides significantly better segmentation accuracies for both tumor and tumor stroma classes. In general, for all methods larger number of annotated slides results in higher accuracies; S–o-M approach achieves competitive accuracies with low number of annotated WSIs. (**C**) Visual comparison of multi-class segmentation accuracy in test slides for resulting final models in three approaches dependent on number of annotated slides used. The review of the images by pathology experts shows higher quality of segmentation for S–o-M approach, especially when lower number of annotated slides was used.

Adding more annotated data increases the similarity of non-annotated slides to annotated ones, meaning improved coverage of data variability and that it might be easier to find matching “morphology expert” model (Fig. 5A; for similarity matrices for independent experiment runs with 5 and 15 annotated WSIs see Suppl. Figures 17 and 18 respectively). Statistical analysis using two-sample t-tests was performed to assess whether increasing the number of annotated WSIs led to statistically significant differences in the distribution of maximum similarity scores across 200 non-annotated WSIs. The results suggest that the increase in similarity is statistically significant, with p-values of 0.00034 (5 vs. 10 annotated WSIs), 0.03922 (10 vs. 15), and 1.77 × 10⁻⁸ (5 vs. 15), all reaching conventional significance levels (p < 0.05). Further, S–o-M outperforms supervised and traditional SSL methods, however, the differences are less prominent when number of annotated slides is 15 (Fig. 5B; for more details including performance below 5 and 15 annotated WSIs on other tissue classes see Suppl. Figures 19 and 20 respectively, and the segmentation performance of the three models under 5 and 15 annotated WSIs for tumor and tumor stroma across various external datasets see Suppl. Figure 21A and 21B, respectively). Similarly to previous experiments, the visual evaluation by pathologists aligns with the quantitative findings, further supporting the effectiveness of the S–o-M approach in all three scenarios concerning number of annotated slides.

### Impact of number of non-annotated WSIs

To assess the effect of non-annotated data volume on method’s performance, we conducted experiments using different numbers of non-annotated WSIs (n = 20, 50, 100, with 200 already tested in our initial setup), while keeping all other settings same as in initial setup (including annotated WSIs, n = 10). Evaluation was performed using five independent test datasets like in the initial setup.

**Fig. 6:**
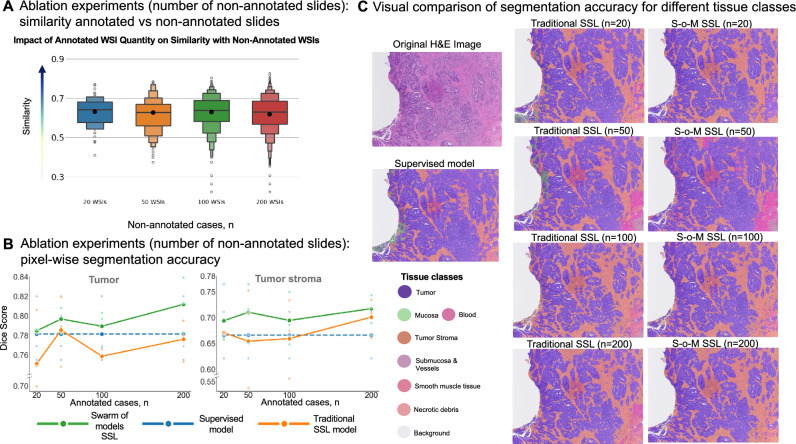
Ablation experiments (number of non-annotated cases): whole-slide image (WSI) similarity analysis, comparison of segmentation accuracy for different approaches, and visual evaluation of segmentation accuracy (**A**) Distribution of case-level similarity values for different numbers non-annotated WSIs (against annotated slides, always same amount, n = 10). During this ablation experiment we evaluate the impact of the number of non-annotated WSIs included in training (TCGA colorectal cancer cohort). Conclusion: similarity values show roughly equal distribution in four groups (non-annotated slides n = 20, 50, 100, and 200). (**B**) Comparison of segmentation accuracy for different training approaches (TCGA dataset, non-annotated slide n = 20, 50, 100, and 200; non-annotated slide n = 10 in all experiments). Three different approaches (supervised learning, traditional SSL, and SSL using S–o-M) are compared concerning pixel-wise segmentation accuracy (measured by Dice Score). Conclusions: new SSL S–o-M approach provides significantly better segmentation accuracies for both tumor and tumor stroma classes in all four training groups. Although there is a trend to higher accuracy with larger number of non-annotated slides, significant fluctuations can be observed that are probably attributed to different proportion of noise related to pseudo-labels of low quality in four training groups. (**C**) Visual comparison of multi-class segmentation accuracy in test slides for resulting final models in three approaches dependent on number of non-annotated slides used. The review of the images by pathology experts shows higher quality of segmentation for S–o-M approach.

Mean similarity scores for non-annotated images against annotated WSIs (Fig. 6A) were similar reinforcing the finding in previous experiments that similarity is a function of the number of annotated slides (compare Fig. 5A). However, as the number of non-annotated WSIs increases, the similarity distribution becomes more dispersed, and more extreme values begin to appear (similarity matrices of the annotated WSIs and the matching results between each non-annotated WSI and its most similar annotated counterpart for three independent experiments are shown in Suppl. Figures 22, 23 and 24). These extreme values are less highly similar cases and more dissimilar outliers (Fig. 6A), which can be valuable for enriching the dataset with rare or underrepresented morphologies. Again, across three replicates of each training using random slide selection, S–o-M-based model consistently outperformed two other approaches for any tested number of non-annotated data (Fig. 6B; for more details including performance under 20, 50 and 100 non-annotated WSIs on other tissue classes see Suppl. Figure 25, 26 and 27, respectively, and the segmentation performance of the three models under 20, 50 and 100 non-annotated WSIs for tumor and tumor stroma across various external datasets see Suppl. Figures 28A, 28B and 28C, respectively). Some fluctuations were observed (higher accuracies for n = 50 compared to n = 100). Detailed review did not reveal any technical issues, and these fluctuations should be attributed to stochastic nature of information included in non-annotated training WSIs, the currently unknown issue related to optimal selection of non-annotated slides for training that warrants additional investigation and was out-of-scope of our study. Visual review by pathologists, similar to previous experiments, validated better segmentation accuracy of S–o-M models (Fig. 6C).

## Discussion

In this study, we introduce a new SSL framework for semantic segmentation tasks in pathology. It addresses the acute problem of very limited annotation data available for training of diagnostic models via using manual annotations by experts (highly laborious job) in the most effective way. Central to our approach is the S–o-M strategy, which leverages inter-slide similarity to assign specific “morphology expert” models from the pool to generate high-quality pseudo-labels for non-annotated training WSIs. Through comprehensive experiments, we demonstrate the robustness and generalizability of this method across varying annotation budgets, different quantities of unlabeled data, and using only single center data for training (less variance and potentially less generalizability compared to using multi-centric data). Notably, our approach consistently outperformed both fully supervised models and conventional SSL frameworks.

### Computational efficiency and practical considerations

Although the S–o-M framework introduces additional computational overhead due to the training of multiple “morphology expert” models and the initial tumor detection model, this cost remains moderate and manageable. All experiments were conducted using an NVIDIA A100 GPU (80 GB). Under the standard configuration with 10 annotated WSIs, the total training time for S–o-M was approximately 42 h, corresponding to 1.19 × the runtime of the traditional SSL baseline, A detailed comparison of computational cost across methods is provided in Suppl. Figure 29. The majority of the additional cost arises from the tumor detection model and the independent training of single-case expert models; however, each model converges quickly because it is trained on a limited number of tiles per case. Moreover, all models were trained with a default setting of 36 epochs, yet in practice, the optimal checkpoints, particularly for the expert models, were typically reached before the final epoch, indicating that the total runtime could be further reduced via early stopping. Despite the slightly higher computational cost, the S–o-M approach yields consistently superior segmentation accuracy and robustness, suggesting that the modest increase in runtime is justified by clear performance gains. Future optimization could focus on integrating lightweight backbone architectures or parameter-efficient fine-tuning methods to further enhance scalability.

### Related work

#### SSL in computer vision

SSL has become a cornerstone in computer vision, particularly for annotation-intensive tasks like semantic segmentation. Several approaches were investigated earlier. The Mean Teacher model^37^ employs an exponential moving average (EMA) of student weights to create a stable teacher, promoting temporal consistency in predictions. FixMatch^38^ builds on pseudo-labeling by combining strong data augmentation with confidence thresholding, achieving state-of-the-art results on natural image benchmarks, though its reliance on fixed thresholds can be brittle in noisy or ambiguous settings. To improve robustness, recent methods have incorporated mechanisms such as uncertainty filtering^39^ and ensemble-based self-label refinement^40^. Contrastive learning has also been introduced to enhance representation quality, e.g., PseCo^41^ couples classification with contrastive losses, while ConMatch^42^ introduces contrastive augmentation to regularize learning. Despite their effectiveness, most of these models are designed for natural images, where label noise is relatively uniform and structure is less complex, limiting their applicability to domains like computational pathology.

#### Semi-supervised learning in computational pathology

Pixel-level annotations in WSIs remains a critical bottleneck in computational pathology due to the size and complexity of tissue structures. SSL has emerged as an attractive solution, enabling models to exploit large volumes of unlabeled data. However, pathology-specific challenges, such as staining variability, inter-class ambiguity (especially between tumor and stroma), and morphological heterogeneity, necessitate domain-adapted SSL strategies^43^.

Due to the difficulty of acquiring high-precision annotations, early SSL efforts primarily focused on slide/patch-level classification. For instance, Shaw et al.^44^ utilized a teacher-student framework for colorectal cancer grading. Peikari et al.^45^ proposed a clustering-guided semi-supervised approach to reduce dependence on labeled data. More recently, Zhang et al.^46^ introduced a dual-teacher contrastive regularization method to enhance classification robustness. While these methods achieved notable results, they fall short in downstream tasks requiring spatial precision, such as tumor–stroma segmentation or tumor microenvironment characterization. To reduce the need for pixel-level labels, Han et al.^47^ proposed a weakly supervised framework combining Multi-layer Pseudo-Supervision (MLPS) and Progressive Dropout Attention (PDA). Their method leverages patch-level classification labels to generate pseudo-masks through CAM-based techniques, significantly lowering annotation costs while achieving performance comparable to fully supervised models.

Beyond CAM-based pseudo-mask generation, several recent studies have explored weakly supervised WSI- or region-level segmentation through scalable sequence- or state-space modeling architectures. For instance, PathMamba^48^ employs selective state-space scanning to capture long-range morphological dependencies, representing a shift from class-activation heuristics toward more structured slide-level sequence modeling. DIPathMamba^49^ extends this approach by investigating domain-incremental weak supervision, demonstrating that segmentation models can adapt across sequential pathology domains via domain-parameter constraints and uncertainty-aware supervision losses. These works highlight an emerging trend of leveraging large unlabeled slide repositories with only weak labels, while attempting to improve cross-domain consistency. However, they remain predominantly weakly supervised and do not utilize targeted expert selection for slide-wise morphological specialization.

In contrast, our proposed S–o-M method is specifically designed to address the challenges of semantic segmentation in pathology. By leveraging inter-slide similarity and expert model guidance to refine pseudo-labels, S–o-M enhances spatial accuracy beyond the capabilities of weakly supervised approaches. This allows for more precise delineation of tumor and stromal regions, making it particularly effective for downstream applications that require fine-grained detection of these compartments^12,50^.

With the emergence of high-precision annotations, pixel-level segmentation using SSL in pathology has started to gain traction. Shi et al.^51^ introduced SSPCL, a semi-supervised pixel contrastive learning framework for histopathological tissue segmentation. SSPCL incorporates both labeled and unlabeled data through domain-specific sampling to model slide-level semantic relationships. While the method effectively enforces local feature alignment by leveraging spatial continuity, its reliance on spatial coherence assumptions may reduce performance when handling highly heterogeneous or rare tumor subtypes with inconsistent structural patterns. Moreover, the computational overhead of pixel-level contrastive learning and memory bank maintenance poses scalability challenges for large WSIs. In contrast, our S–o-M method circumvents the reliance on spatial coherence by comparing representative features at the slide level to estimate inter-slide similarity, making it more adaptable to diverse tumor subtypes and less computationally demanding.

TS-Net, a convolution-transformer hybrid model designed for semi-supervised tissue segmentation, demonstrated promising results^52^. However, its effectiveness is contingent upon the representativeness of the unlabeled data, and the lack of external validation (e.g., on multi-center datasets) limits conclusions about its generalizability. In contrast, our S–o-M method was rigorously evaluated using five independent external datasets, demonstrating strong generalization across diverse clinical sources and improved robustness to domain variability. This extensive validation highlights the scalability and adaptability of our approach in real-world diagnostic settings.

Lai et al. proposed a joint semi-supervised and active learning framework for gigapixel pathology image segmentation, aiming to minimize annotation efforts. Their method integrates region-based active learning with SSL, achieving competitive results while labeling only 0.1% of the data^53^. However, the reliance on iterative expert annotation of uncertain regions may significantly limit scalability, particularly in multi-institutional contexts due to expert availability and inter-observer variability. In contrast to methods, which depend on iterative expert annotation during active learning cycles, our S–o-M method significantly reduces the reliance on manual labeling. By using a small set of less expert-annotated WSIs combined with 200 non-annotated WSIs, our method can achieve strong performance on multi-institutional external datasets. This approach not only mitigates the challenges posed by necessity of (real-time) expert availability in active learning frameworks but also demonstrates scalability, making it suitable for real-world, resource-constrained settings where expert annotations may be limited.

Shin et al. proposed a graph-based pseudo-labeling framework for semi-supervised pathology image classification, which refines pseudo-labels via graph segmentation by modeling local and global contextual relationships between tissue patches^54^. While this approach improves label coherence through topological constraints, its performance may degrade when initial network predictions, used as seed labels, are highly uncertain or noisy, especially under extremely limited labeled data conditions. By leveraging expert-informed pseudo-labeling and inter-slide similarity, our method can reduce reliance on potentially noisy seed labels and ensure more reliable label refinement.

Fouad et al. presented a hybrid strategy combining unsupervised superpixel-based consensus clustering with a self-training semi-supervised classifier (Random Forest) for epithelium-stroma segmentation ^55^. Although interpretable and effective with minimal labeled data, the reliance on handcrafted features and conventional machine learning techniques limits both scalability and representational capacity relative to modern deep learning models. And Our S–o-M method utilizes deep learning models, which offer significantly improved scalability and representational capacity. By harnessing the power of modern neural networks, our method avoids the limitations of handcrafted features and is better suited to handle complex pathology tasks, even with minimal labeled data.

Recent studies have explored complementary directions that align with our framework. ESASeg^56^ mitigates expression-site variability in IHC images by combining self-supervised pretraining with domain adaptation. Specifically, a multi-level semantic feature alignment strategy, together with a pathology-aware self-supervised task (resolution prediction), yields expression-site-invariant representations and enhances tumor segmentation across domains. Another line of work focuses on uncertainty-aware collaborative learning: a global-attention GNN equipped with Bayesian collaborative learning (BCL)^57^ jointly models local and global context while optimizing graph- and patch-level classifiers, thereby improving robustness under semantic ambiguity. In addition, registration-enhanced weak supervision (RMIL)^58^ leverages inter-slice registration to propagate labels across neighboring sections and augment weak annotations, resulting in more reliable MIL-based WSI classification. Collectively, these methodologies, domain adaptation, Bayesian uncertainty modeling, and registration-based pseudo-label refinement, complement the S–o-M paradigm and highlight practical strategies for improving pseudo-label reliability and cross-site generalization.

Beyond conventional semi-supervised segmentation, recent advances in computational pathology have explored self-supervised pretraining, contrastive learning combined with pseudo-labeling, and federated SSL frameworks. For example, DINO-based feature extractors have been shown to outperform ImageNet initialization on kidney biopsy images^59^, whereas hierarchical ViT models such as CypherViT capture multi-scale phenotypes through multi-token self-supervision^60^. Federated semi-supervised segmentation approaches with pseudo-label denoising have further addressed cross-site generalization and privacy concerns^61^. The S–o-M framework complements these approaches by emphasizing similarity-guided pseudo-label refinement, leveraging a small set of expert-annotated slides to guide large unlabeled datasets.

### Limitations and further directions

In scenarios with very few annotated WSIs, such as using 5 annotated slides alongside 200 non-annotated WSIs, both the S–o-M framework and traditional pseudo-label-based SSL underperformed compared to a supervised model trained solely on the annotated slides (Fig. 5). A likely contributing factor is the imbalance between a small annotated pool and a large unlabeled pool: most non-annotated WSIs exhibit low similarity to any annotated slide, with similarity scores predominantly below 0.6 (Suppl. Figure 17). This limits the reliability of assigned experts and weakens pseudo-label quality, particularly for tumor and tumor stroma regions exhibiting substantial inter- and intra-tumoral morphological heterogeneity. These results highlight a potential limitation of S–o-M under extremely low-annotation, high-unlabeled conditions, and suggest that increasing annotated slide diversity or filtering unlabeled slides based on similarity thresholds may alleviate this issue.

Although our proposed method consistently improves segmentation performance across different dataset configurations, it remains sensitive to the selection of annotated cases. Choosing representative slides for annotation requires input from experienced pathologists, which introduces some manual overhead. However, this burden can be significantly reduced using a high-accuracy tumor segmentation model in combination with our similarity analysis pipeline, which aids in identifying diverse and informative cases for annotation.

Notably, the architecture of our method, based on a S–o-M and a shared annotated feature pool, makes it well-suited for federated learning. In medical imaging, where patient privacy concerns and data protection regulations often limit centralized data collection, federated learning has become increasingly important. Our approach avoids the need to share raw slide data across institutions; instead, only lightweight components such as model parameters or extracted features from annotated slides need to be shared. This design supports collaborative learning across institutions while preserving data privacy.

Looking ahead, further improvements could focus on automating the case selection process through clustering or active learning, reducing the reliance on expert curation. When combined with federated infrastructure, our similarity-guided semi-supervised framework offers a scalable and privacy-preserving solution for clinical deployment in real-world pathology workflows.

## Conclusion

In this study, we proposed a novel similarity-guided SSL framework that integrates morphology expert model selection from a S–o-M to enhance pseudo-label generation for tumor and tumor stroma segmentation in histopathology. Through comprehensive ablation experiments, we demonstrated the robustness of our method across varying quantities of annotated and unannotated data, as well as across different dataset compositions. Our approach consistently outperformed both traditional pseudo-labeling and supervised frameworks particularly in low-annotation scenarios. Moreover, its architecture aligns naturally with federated learning, offering a privacy-preserving solution that avoids direct data sharing, an important consideration in medical imaging. These results underscore the potential of incorporating similarity information to improve the reliability, adaptability, and scalability of SSL in computational pathology.

## Supplementary Information

Supplementary Information 1.

Supplementary Information 2.

## Data Availability

The results here are in whole or part based upon data generated by the TCGA Research Network: https://www.cancer.gov/tcga
