# Supplementary material for: Similarity-guided swarm of models: enhancing semi-supervised learning in computational pathology
Source: Sci Rep. 2025 Dec 30;15:45667. doi: 10.1038/s41598-025-33281-3 (PMC12753758; doi:10.1038/s41598-025-33281-3)
Supplement: Supplementary file 1 — Supplementary Information 1. [file 41598_2025_33281_MOESM1_ESM.pdf]

Algorithm 1. S-o-M Semi-Supervised Segmentation Framework

Example configuration: 10 annotated WSIs and 200 non-annotated WSIs.

Input:

- Annotated WSIs $\mathcal{D}_{ann} = \{A_1, A_2, \ldots, A_{10}\}$
- Non-annotated WSIs $\mathcal{D}_{unl} = \{U_1, U_2, \ldots, U_{200}\}$
- Foundation model F_{enc} (e.g., UNI)
- Tumor content threshold T_{tumor}

Output:

- Final semi-supervised segmentation model M_{final}

Procedure:

1. **Feature Extraction for Annotated WSIs**
   For each annotated slide $A_i \epsilon\ \mathcal{D}_{ann}$:
   1.1 Extract tiles covering annotated tumor regions.
   1.2 Obtain tile embeddings using foundation encoder F_{enc} .
   1.3 Apply K-means clustering (*n* = *5*) to select five representative cluster centroids as slide-level features.
   1.4 Store the five representative feature vectors locally.
2. **Model Training**
   Train the following models using the annotated dataset:
   2.1 A supervised segmentation model M_{sup} .
   2.2 A tumor detection model M_{tumor} .
   2.3 A set of morphology expert models $= \{E_1, E_2, \ldots, E_{10}\}$, each trained exclusively on one annotated WSI.
3. **Pre-filtering of Non-Annotated WSIs**
   For each non-annotated slide $U_j \epsilon\ \mathcal{D}_{unl}$:
   3.1 Divide the WSI into tiles.
   3.2 Apply M_{tumor} to estimate tumor proportion per tile.
   3.3 Retain only tiles where tumor content > T_{tumor}.
4. **Similarity Assessment and Expert Selection**
   For each U_j :
   4.1 Extract 5 representative features using F_{enc} and K-means (*n* = *5*).
   4.2 Compute cosine similarity between its 5 features and those of each A_i.
   4.3 Select the morphology expert E_k corresponding to the annotated slide with the highest mean similarity score.
5. **Pseudo-Label Generation**
   For each tile of U_j :
   5.1 Use E_k to predict tumor and tumor stroma regions.
   5.2 Use M_{sup} to predict remaining tissue classes.
   5.3 Fuse predictions at the tile level to form the final pseudo-label mask.
   5.4 Resolve class overlap by prioritizing tumor/stroma predictions from E_k.
6. **Quality Control**
   Apply the pre-trained QC model M_{qc} (e.g.. GrandQC) to detect artifacts;
   set corresponding mask regions to zero (ignored during training).
7. **Final Model Training**
   Merge annotated and pseudo-labeled datasets and train the final semi-supervised segmentation model M_{final}.

Scalability Discussion:
The computational complexity of the S-o-M framework scales approximately linearly with the number of annotated WSIs (N_{ann}). Each annotated WSI corresponds to one morphology expert model (E_i), leading to $O(N_{ann})$ training cost for experts. However, since each expert is trained on a limited number of tiles from a single slide, individual training runs are lightweight and can be parallelized across GPUs.
During inference on non-annotated WSIs, the similarity computation requires only $5{\times}5{\times}N_{ann}$ cosine operations (using 5 representative features per slide), which remains negligible even for larger N_{ann}. Therefore, both training and inference costs increase modestly and predictably with the number of annotated slides, supporting practical scalability of the framework.

Supplementary Fig. 1 This figure illustrates the detailed step-by-step workflow of the S-o-M framework, including input, output, Procedure and Scalability Discussion.

Similarity Analysis Among Annotated Cases Using cosine similarity-metric with Prov-GigaPath

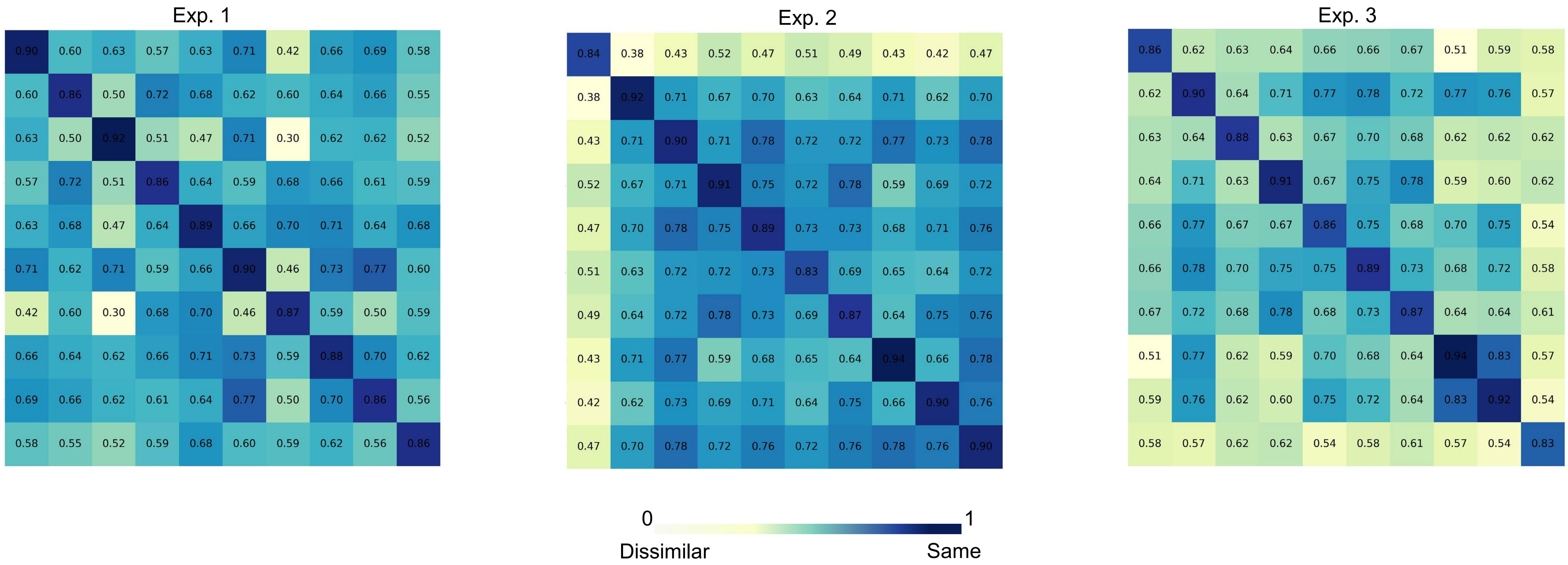

Supplementary Fig. 2 Three heatmaps presents the similarity analysis using the initial dataset setup with three different selections of 10 annotated cases. The similarity is computed using Prov-GigaPath as the feature extractor (encoder). Darker colors indicate higher similarity between cases.

Impact of Cluster Number on Segmentation Performance (Exp. 1)

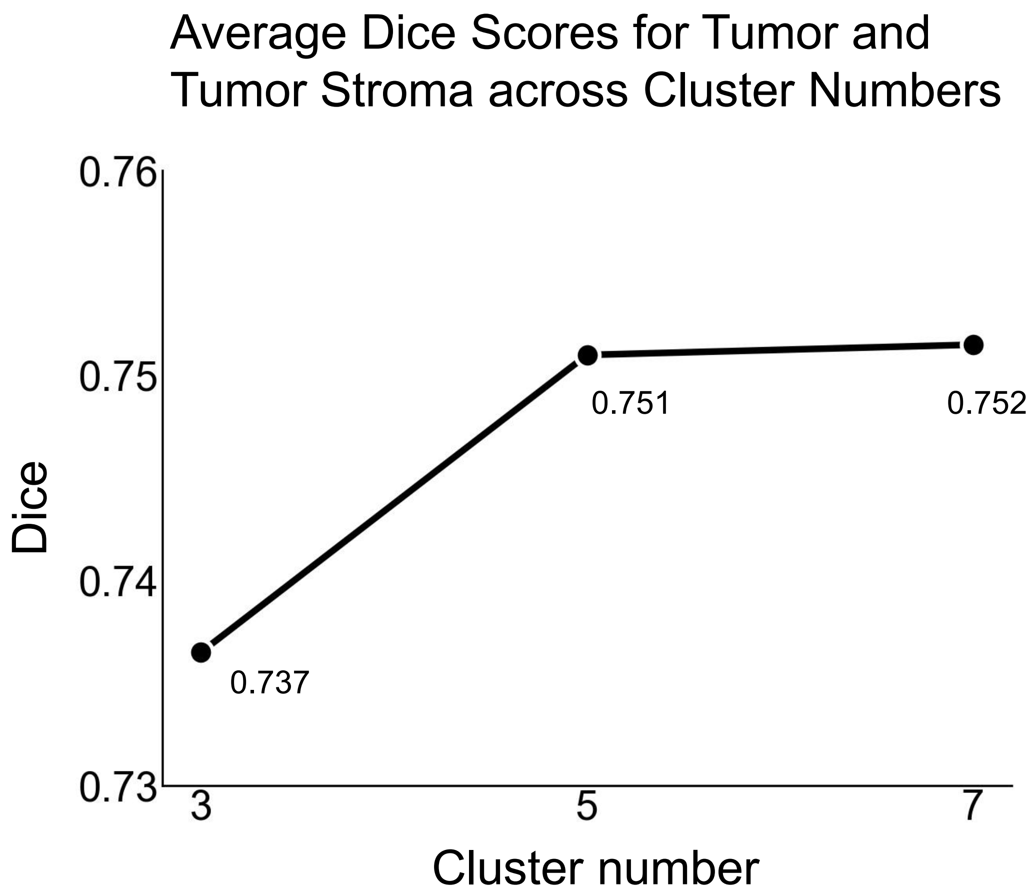

Supplementary Fig. 3 This figure illustrates the effect of different cluster numbers in K-Means on segmentation performance measured by Dice score: *Average Dice scores for both tumor and tumor stroma across varying cluster numbers*. The results show how varying the cluster number influences segmentation.

Effect of Cluster Number on Feature Similarity between Annotated and Non-annotated Cases (Exp. 1)

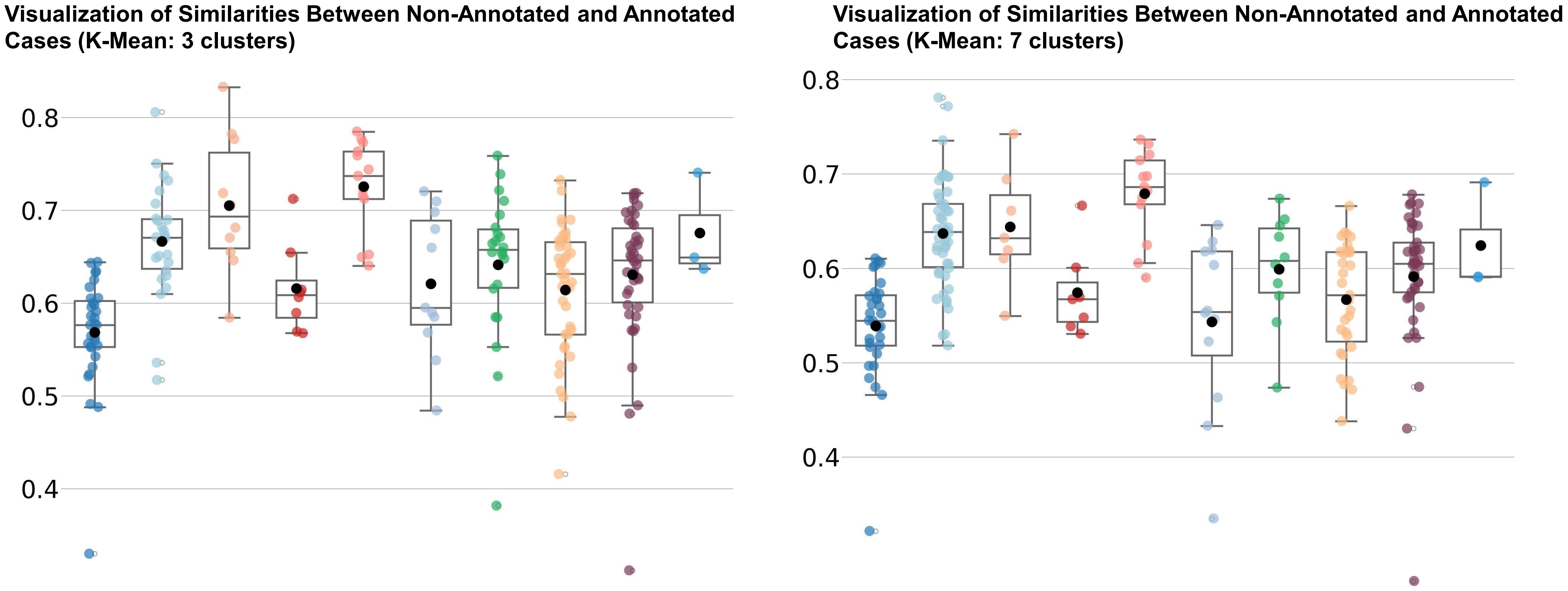

Supplementary Fig. 4 This figure presents the feature similarity between non-annotated and annotated cases using different numbers of clusters in K-Means. A smaller cluster number (*K = 3*) produces coarser WSI representations, compressing more patches into fewer centers and reducing subtle differences between WSIs, which increases the average similarity of non-annotated samples with annotated ones. A larger cluster number (*K = 7*) preserves finer distinctions, yielding more discriminative similarity patterns.

Similarity Analysis Among Annotated Cases Using L1 Distance with UNI

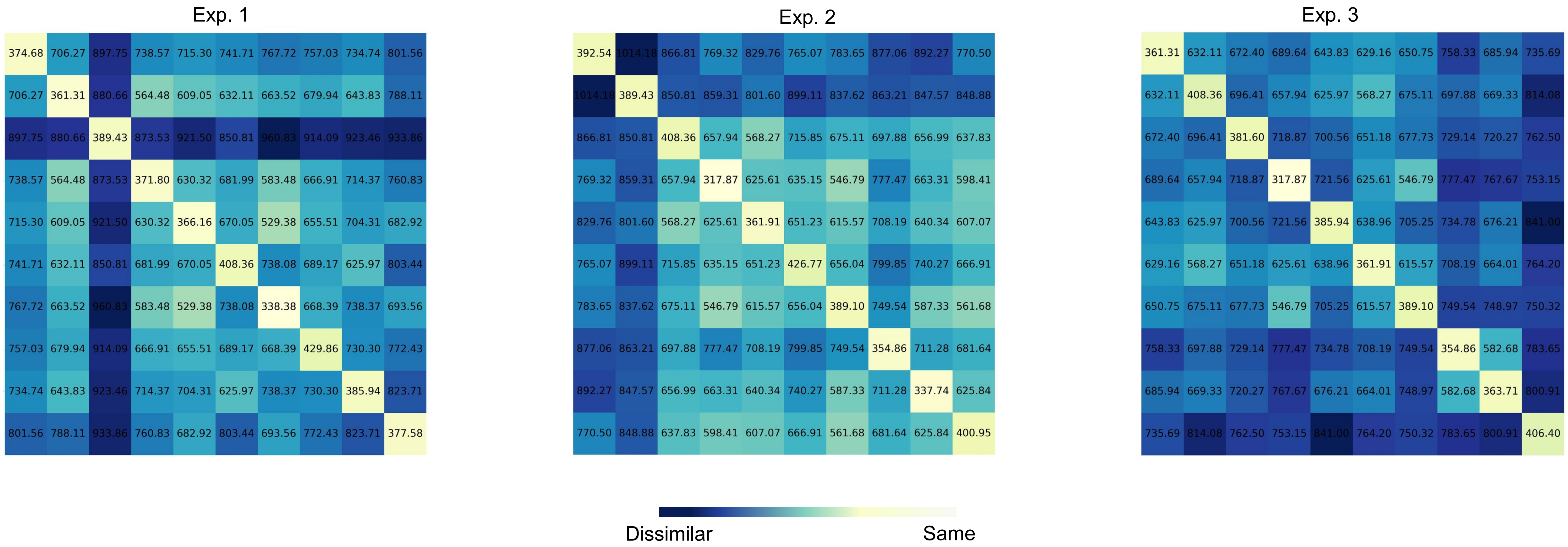

Supplementary Fig. 5 This figure presents three heatmaps illustrating the similarity analysis using the initial dataset setup with three different selections of 10 annotated cases. The similarity is computed using the L1 Distance, where lighter colors indicate higher similarity between cases

Similarity Analysis Among Annotated Cases Using L2 Distance with UNI

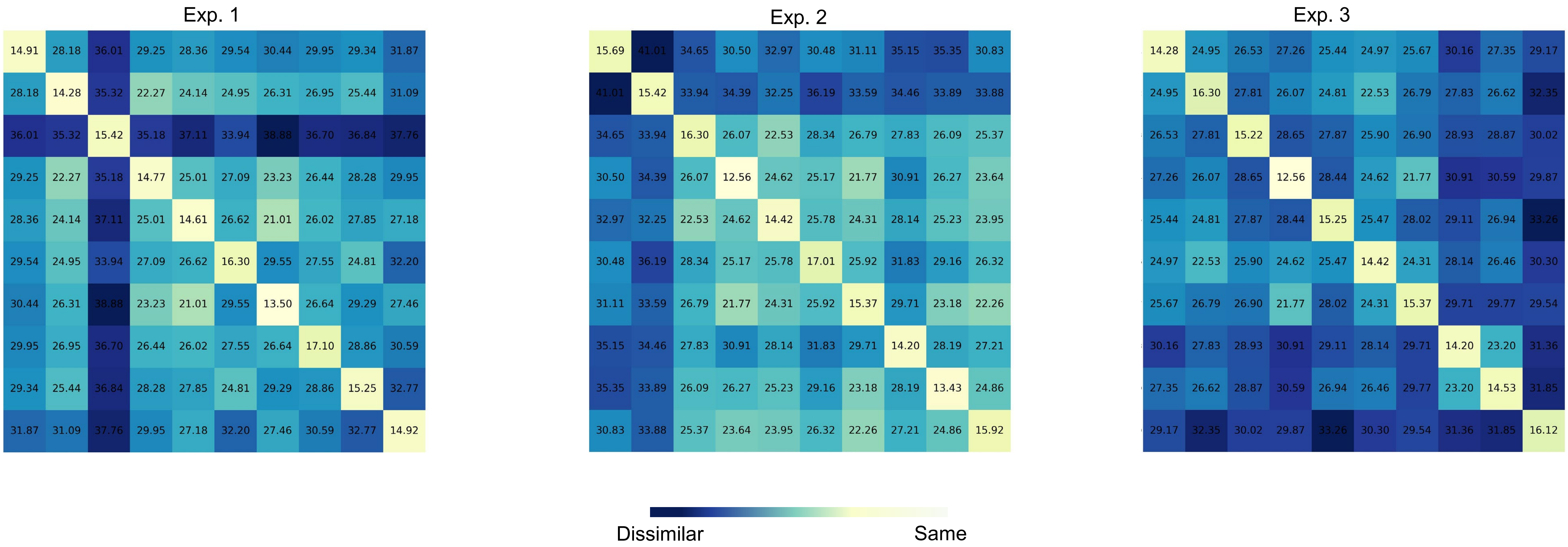

Supplementary Fig. 6 This figure presents three heatmaps illustrating the similarity analysis using the initial dataset setup with three different selections of 10 annotated cases. The similarity is computed using the L2 Distance, where lighter colors indicate higher similarity between cases

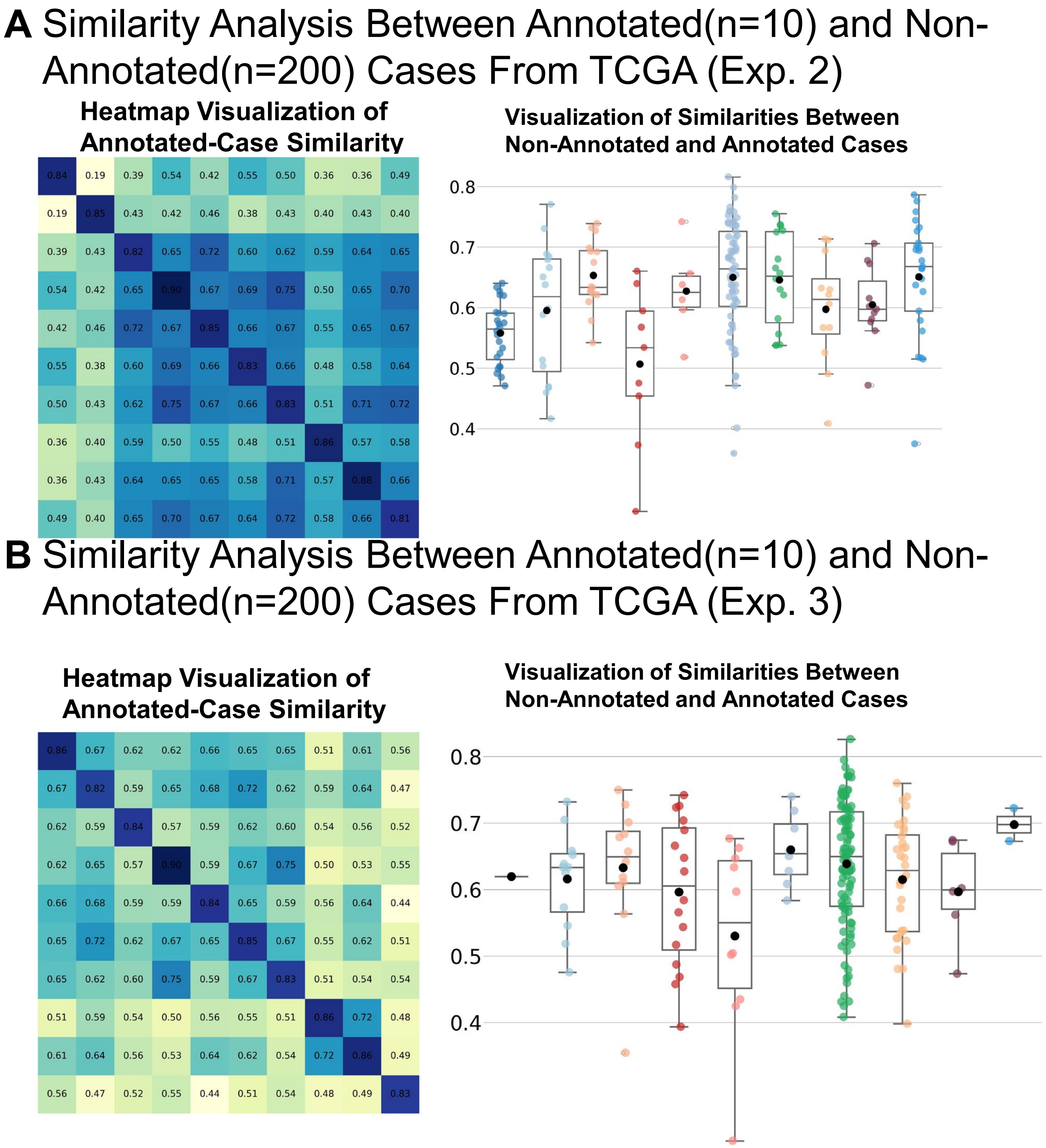

Supplementary Fig. 7 This figure presents the similarity analysis using the initial dataset setup with two different selections of 10 annotated cases. Two heatmaps visualize the similarity between annotated cases in each selection. Two boxplots illustrate the similarity scores between annotated and non-annotated cases, showing which non-annotated cases are most similar to specific annotated cases in each selection.

Dice Score Performance of the Dataset: Annotated(n=10) and Non-Annotated(n=200) Cases From TCGA

Exp. 1

Model Type	Tumor	Tumor Stroma	Benign Mucosa	Submuc	MUSC PROP / MUC	Vessel	Lymph	Necrosis	Blood	Mucin	Back Ground	Average
Tumor Detection	0.858	0.875									0.903	0.879
Supervised Learning	0.755	0.621	0.885	0.727	0.683	0.837	0.906	0.618	0.709	0.664	0.846	0.750
Traditional SSL	0.795	0.660	0.916	0.746	0.805	0.830	0.945	0.662	0.821	0.772	0.864	0.801
Swarm Method	0.813	0.689	0.711	0.688	0.636	0.847	0.804	0.588	0.718	0.759	0.868	0.738

Exp. 2

Model Type	Tumor	Tumor Stroma	Benign Mucosa	Submuc	MUSC PROP / MUC	Vessel	Lymph	Necrosis	Blood	Mucin	Back Ground	Average
Tumor Detection	0.835	0.865									0.884	0.861
Supervised Learning	0.820	0.711	0.895	0.697	0.866	0.745	0	0.752	0.769	0.682	0.780	0.702
Traditional SSL	0.780	0.734	0.882	0.750	0.904	0.749	0	0.771	0.845	0.719	0.762	0.718
Swarm Method	0.839	0.743	0.906	0.767	0.887	0.775	0	0.764	0.861	0.764	0.785	0.736

Exp. 3

Model Type	Tumor	Tumor Stroma	Benign Mucosa	Submuc	MUSC PROP / MUC	Vessel	Lymph	Necrosis	Blood	Mucin	Back Ground	Average
Tumor Detection	0.847	0.867									0.888	0.867
Supervised Learning	0.769	0.666	0.848	0.726	0.750	0.728	0.860	0.846	0.695	0.728	0.765	0.762
Traditional SSL	0.753	0.708	0.883	0.767	0.770	0.777	0.912	0.866	0.801	0.784	0.762	0.799
Swarm Method	0.783	0.719	0.689	0.652	0.779	0.759	0.886	0.869	0.802	0.753	0.828	0.774

Supplementary Fig. 8 This figure presents three tables comparing the Dice scores of different models—Tumor Detection Model, Supervised Learning Model, Traditional SSL Model, and SSL Model with Swarm Models—under the initial dataset setup. Each table corresponds to a different selection of 10 annotated cases

Performance of Tumor-Detection

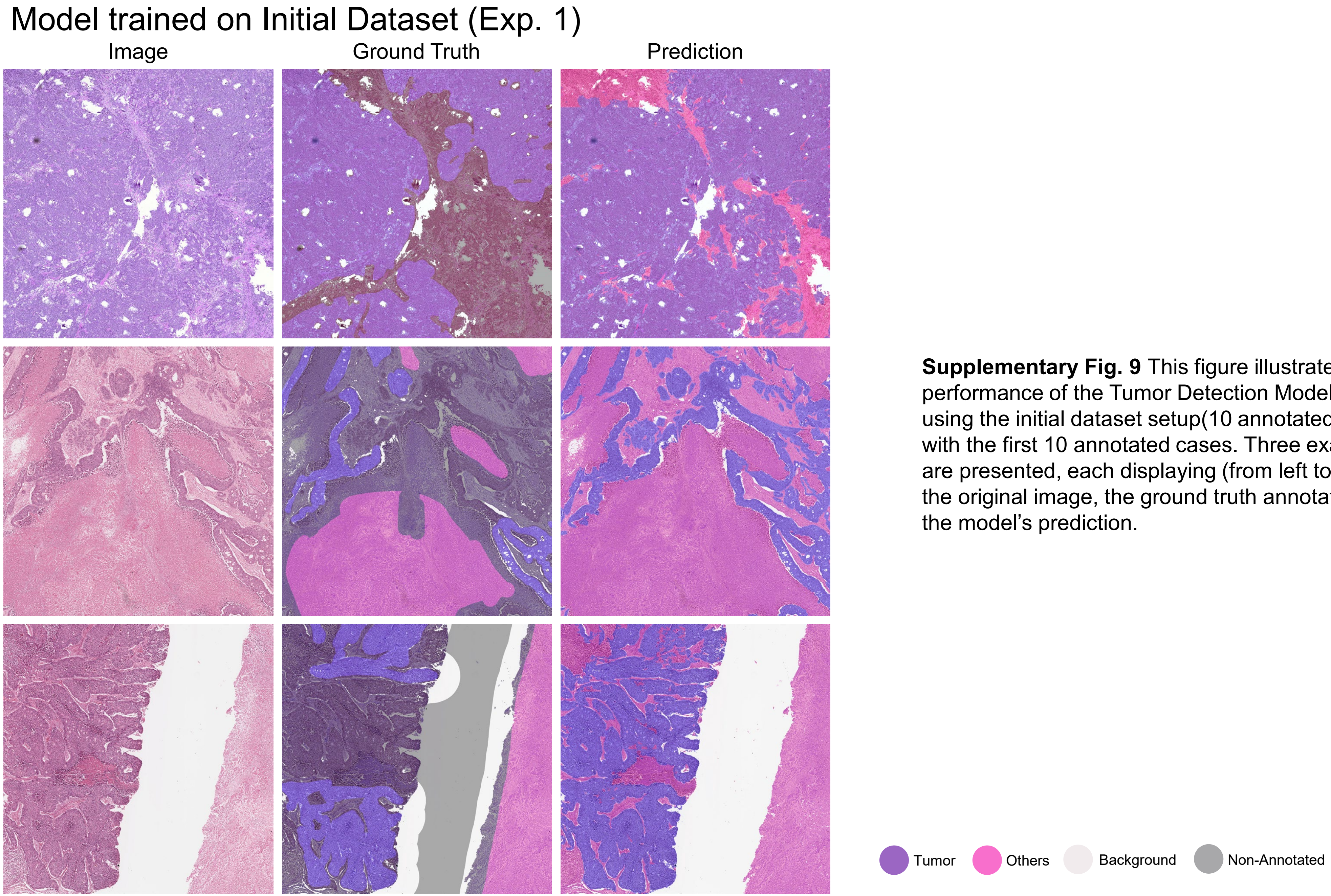

Supplementary Fig. 9 This figure illustrates the performance of the Tumor Detection Model, trained using the initial dataset setup(10 annotated case) with the first 10 annotated cases. Three examples are presented, each displaying (from left to right): the original image, the ground truth annotation, and the model's prediction.

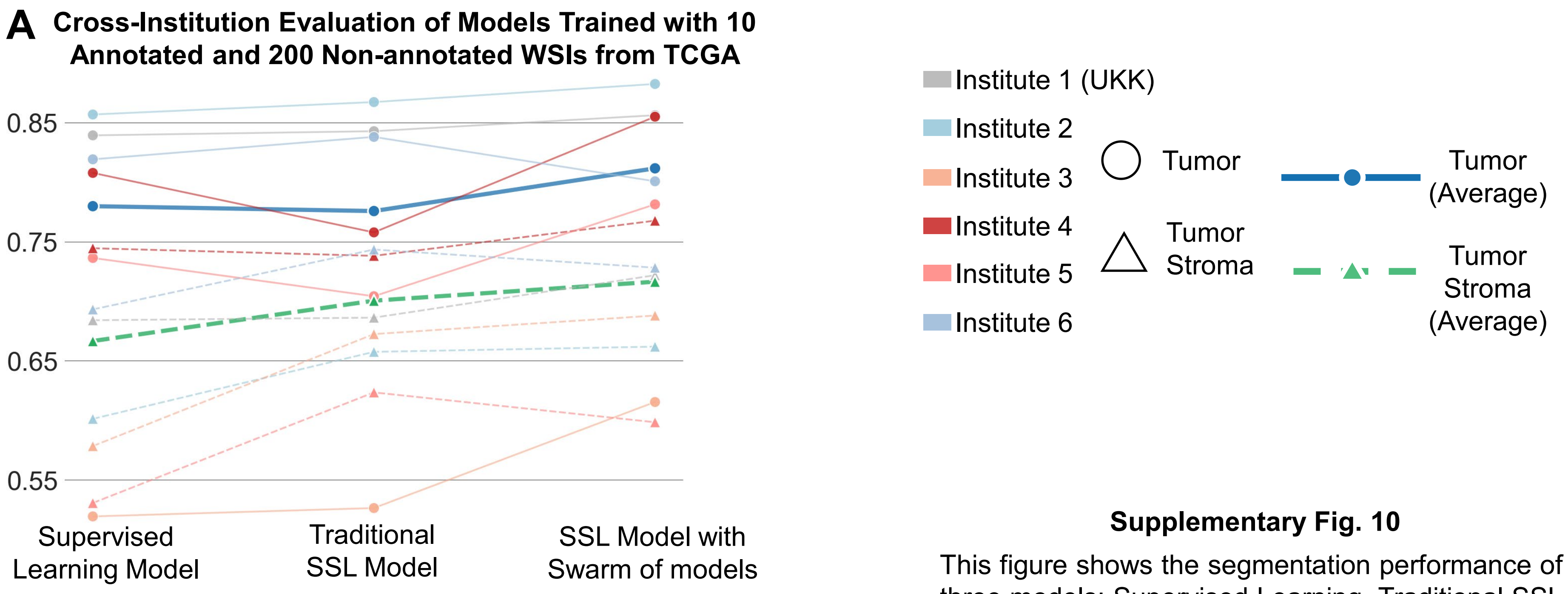

Supplementary Fig. 10

This figure shows the segmentation performance of three models: Supervised Learning, Traditional SSL, and SSL with Swarm Models on tumor and tumor stroma across test datasets from CRAG dataset and four independent institutes. All models were trained using the TCGA dataset. The comparison highlights the generalizability and robustness of each model when applied to external, multi-institutional data.

Recall and Precision for Tumor and Tumor Stroma in Initial Experiments and Single-Center Ablation Study (Exp.2)

	**Recall**		**Precision**	
	Tumor	Tumor Stroma	Tumor	Tumor Stroma
initial experiment	0.843	0.879	0.701	0.672
single-center ablation	0.861	0.898	0.640	0.569

Supplementary Fig. 11 Recall and Precision for Tumor and Tumor Stroma segmentation. Results are reported for the initial experimental setup (10 annotated WSIs with 200 non-annotated WSIs, TCGA) and for the single-center ablation configurations (10 annotated WSIs with 200 non-annotated WSIs, UKK).

Segmentation Performance Comparison across Different Methods (Exp. 1)

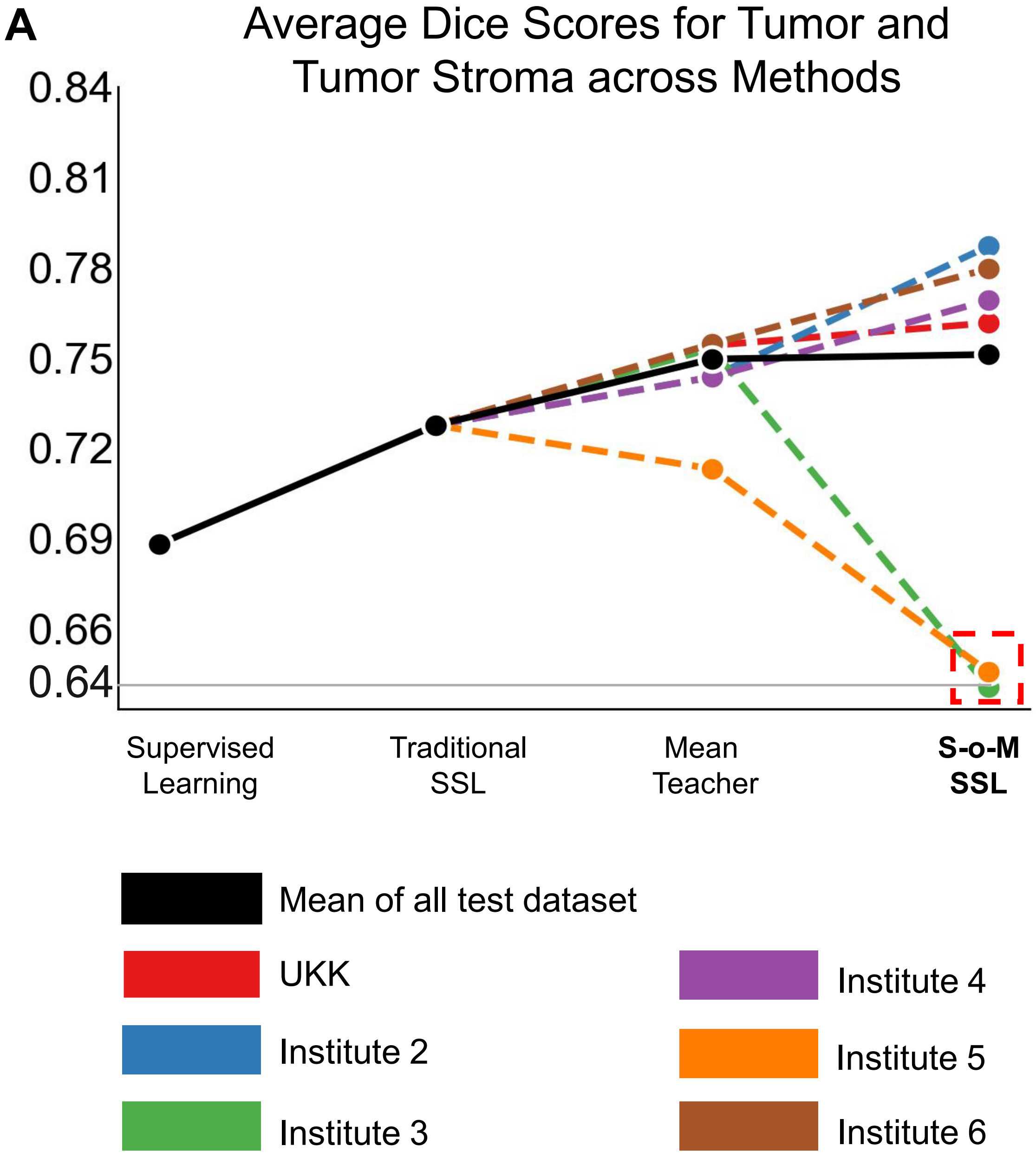

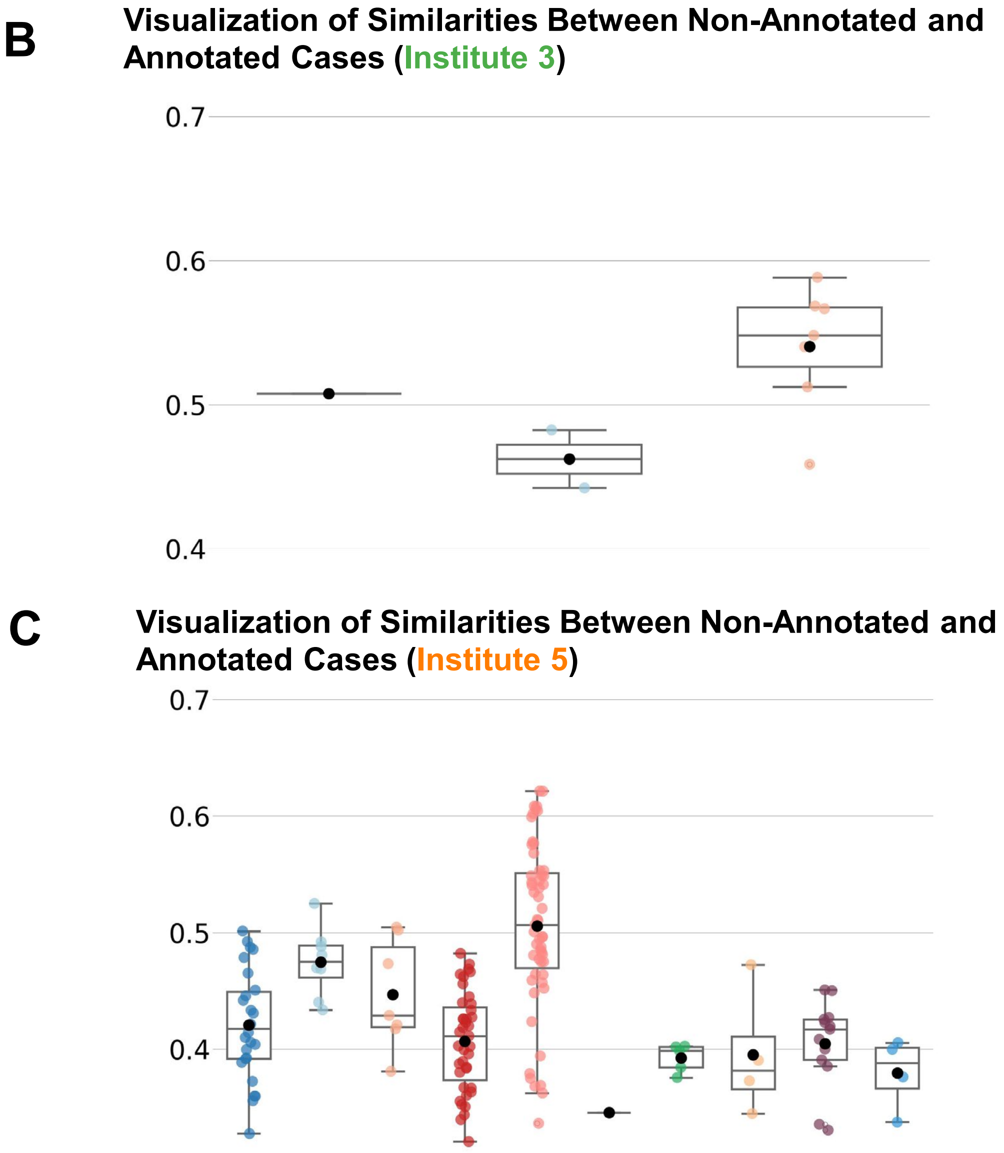

Supplementary Fig. 12 This figure presents cross-institute segmentation robustness. (A) Average Dice scores for tumor and tumor stroma across methods, where institutes 3 and 5 show notably lower performance. (B–C) Visualization of similarities between non-annotated and annotated cases for institutes 3 and 5, demonstrating significantly lower similarity levels, consistent with their reduced segmentation performance.

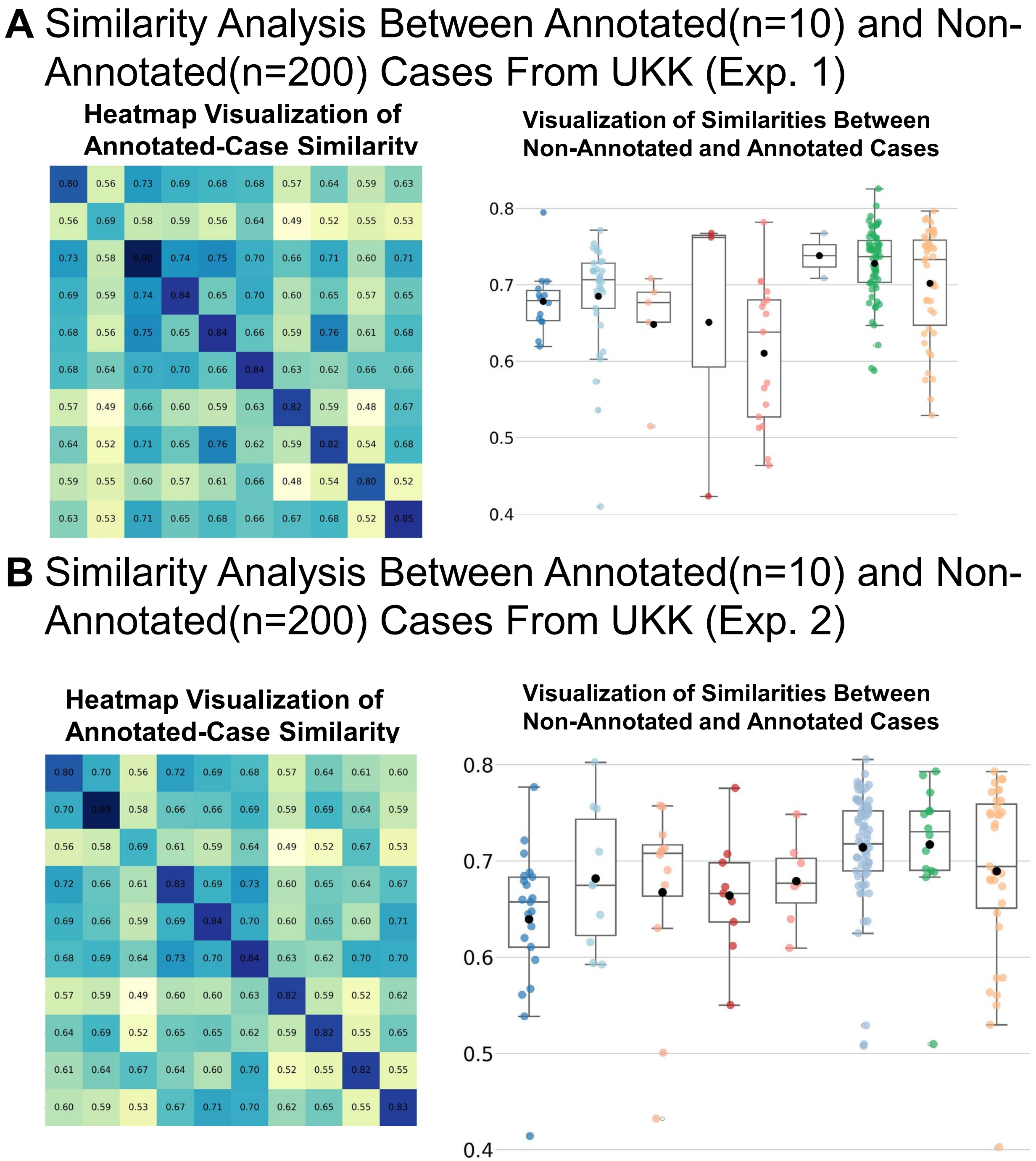

Supplementary Fig. 13 This figure presents the similarity analysis using a dataset setup of 10 annotated cases and 200 non-annotated cases from UKK, with two different selections of 10 annotated cases. Two heatmaps visualize the similarity between annotated cases in each selection. Two boxplots illustrate the similarity scores between annotated and non-annotated cases, showing which non-annotated cases are most similar to specific annotated cases in each selection.

Dice Score Performance of the Dataset: Annotated(n=10) and Non-Annotated(n=200) Cases From UKK

Exp. 1

<table>
<tr><th>Model Type</th><th>Tumor</th><th>Tumor Stroma</th><th>Benign Mucosa</th><th>Submuc</th><th>MUSC PROP / MUC</th><th>Vessel</th><th>Lymph</th><th>Necrosis</th><th>Blood</th><th>Mucin</th><th>Back Ground</th><th>Average</th></tr>
<tr><td>Tumor Detection</td><td>0.915</td><td colspan="9">0.884</td><td>0.899</td><td>0.899</td></tr>
<tr><td>Supervised Learning</td><td>0.817</td><td>0.763</td><td>0.851</td><td>0.546</td><td>0.873</td><td>0.660</td><td>0</td><td>0.823</td><td>0</td><td>0.715</td><td>0.817</td><td>0.624</td></tr>
<tr><td>Traditional SSL</td><td>0.832</td><td>0.795</td><td>0.864</td><td>0.681</td><td>0.875</td><td>0.611</td><td>0</td><td>0.840</td><td>0</td><td>0.787</td><td>0.849</td><td>0.649</td></tr>
<tr><td>Swarm Method</td><td>0.869</td><td>0.810</td><td>0.818</td><td>0.640</td><td>0.856</td><td>0.594</td><td>0</td><td>0.845</td><td>0</td><td>0.777</td><td>0.852</td><td>0.642</td></tr>
</table>

Exp. 2

<table>
<tr><th>Model Type</th><th>Tumor</th><th>Tumor Stroma</th><th>Benign Mucosa</th><th>Submuc</th><th>MUSC PROP / MUC</th><th>Vessel</th><th>Lymph</th><th>Necrosis</th><th>Blood</th><th>Mucin</th><th>Back Ground</th><th>Average</th></tr>
<tr><td>Tumor Detection</td><td>0.913</td><td colspan="9">0.884</td><td>0.898</td><td>0.898</td></tr>
<tr><td>Supervised Learning</td><td>0.886</td><td>0.803</td><td>0.849</td><td>0.781</td><td>0.904</td><td>0.691</td><td>0.889</td><td>0</td><td>0</td><td>0.748</td><td>0.858</td><td>0.673</td></tr>
<tr><td>Traditional SSL</td><td>0.887</td><td>0.807</td><td>0.866</td><td>0.811</td><td>0.913</td><td>0.723</td><td>0.912</td><td>0</td><td>0</td><td>0.779</td><td>0.876</td><td>0.688</td></tr>
<tr><td>Swarm Method</td><td>0.892</td><td>0.791</td><td>0.843</td><td>0.777</td><td>0.891</td><td>0.688</td><td>0.906</td><td>0</td><td>0</td><td>0.817</td><td>0.872</td><td>0.680</td></tr>
</table>

Exp. 3

<table>
<tr><th>Model Type</th><th>Tumor</th><th>Tumor Stroma</th><th>Benign Mucosa</th><th>Submuc</th><th>MUSC PROP / MUC</th><th>Vessel</th><th>Lymph</th><th>Necrosis</th><th>Blood</th><th>Mucin</th><th>Back Ground</th><th>Average</th></tr>
<tr><td>Tumor Detection</td><td>0.868</td><td colspan="9">0.883</td><td>0.887</td><td>0.879</td></tr>
<tr><td>Supervised Learning</td><td>0.800</td><td>0.773</td><td>0.889</td><td>0.690</td><td>0.880</td><td>0.625</td><td>0</td><td>0.753</td><td>0</td><td>0.743</td><td>0.852</td><td>0.637</td></tr>
<tr><td>Traditional SSL</td><td>0.822</td><td>0.787</td><td>0.899</td><td>0.744</td><td>0.891</td><td>0.669</td><td>0</td><td>0.814</td><td>0</td><td>0.770</td><td>0.878</td><td>0.661</td></tr>
<tr><td>Swarm Method</td><td>0.887</td><td>0.771</td><td>0.887</td><td>0.724</td><td>0.893</td><td>0.655</td><td>0</td><td>0.827</td><td>0</td><td>0.753</td><td>0.877</td><td>0.661</td></tr>
</table>

Supplementary Fig. 14 This figure presents three tables comparing the Dice scores of different models—Tumor Detection Model, Supervised Learning Model, Traditional SSL Model, and SSL Model with Swarm Models—under a dataset setup of 10 annotated cases and 200 non-annotated cases from UKK. Each table corresponds to a different selection of 10 annotated cases

Performance of Tumor-Detection

Model trained on Dataset from UKK (Exp. 1)

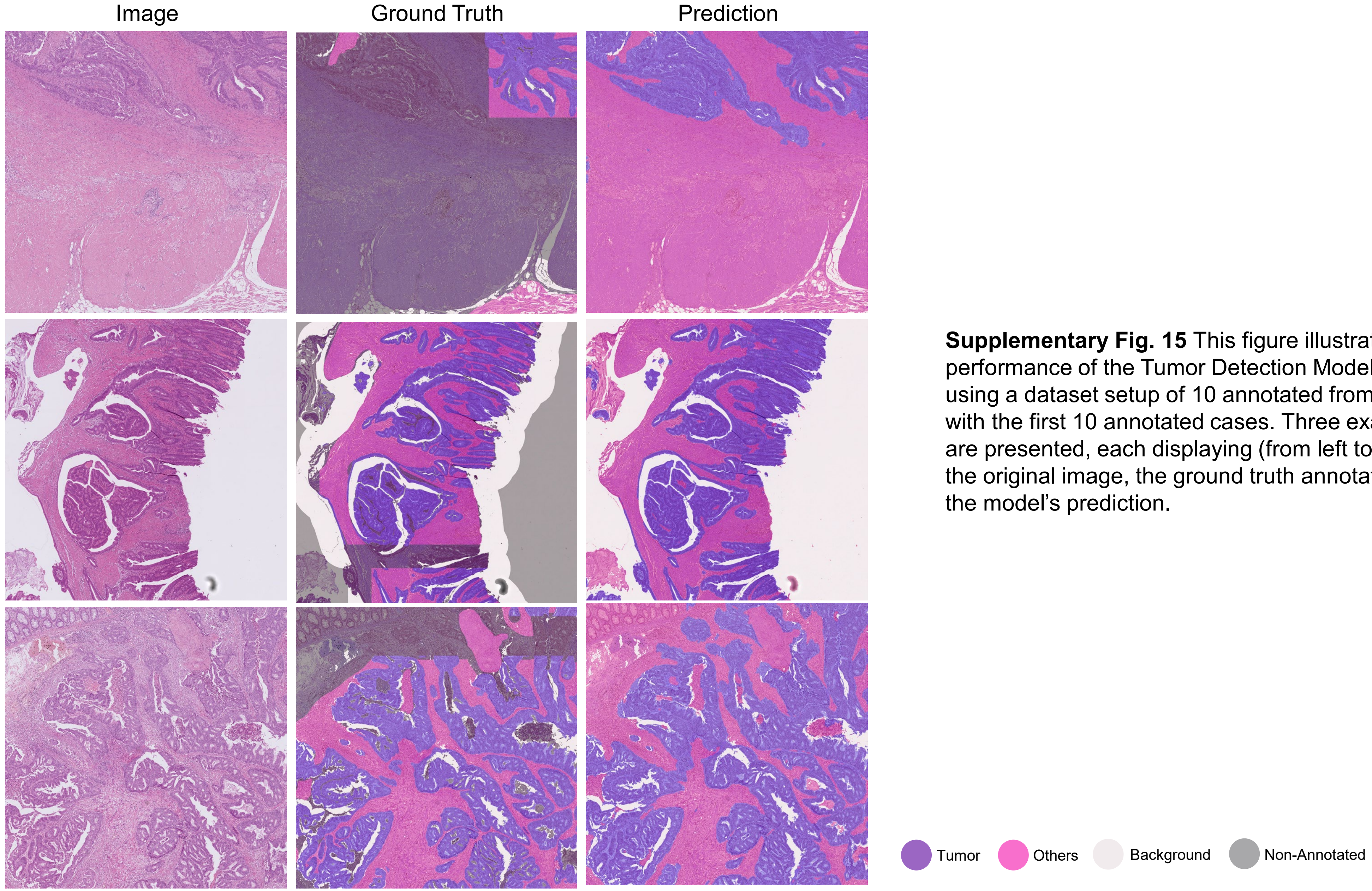

Supplementary Fig. 15 This figure illustrates the performance of the Tumor Detection Model, trained using a dataset setup of 10 annotated from UKK with the first 10 annotated cases. Three examples are presented, each displaying (from left to right): the original image, the ground truth annotation, and the model's prediction.

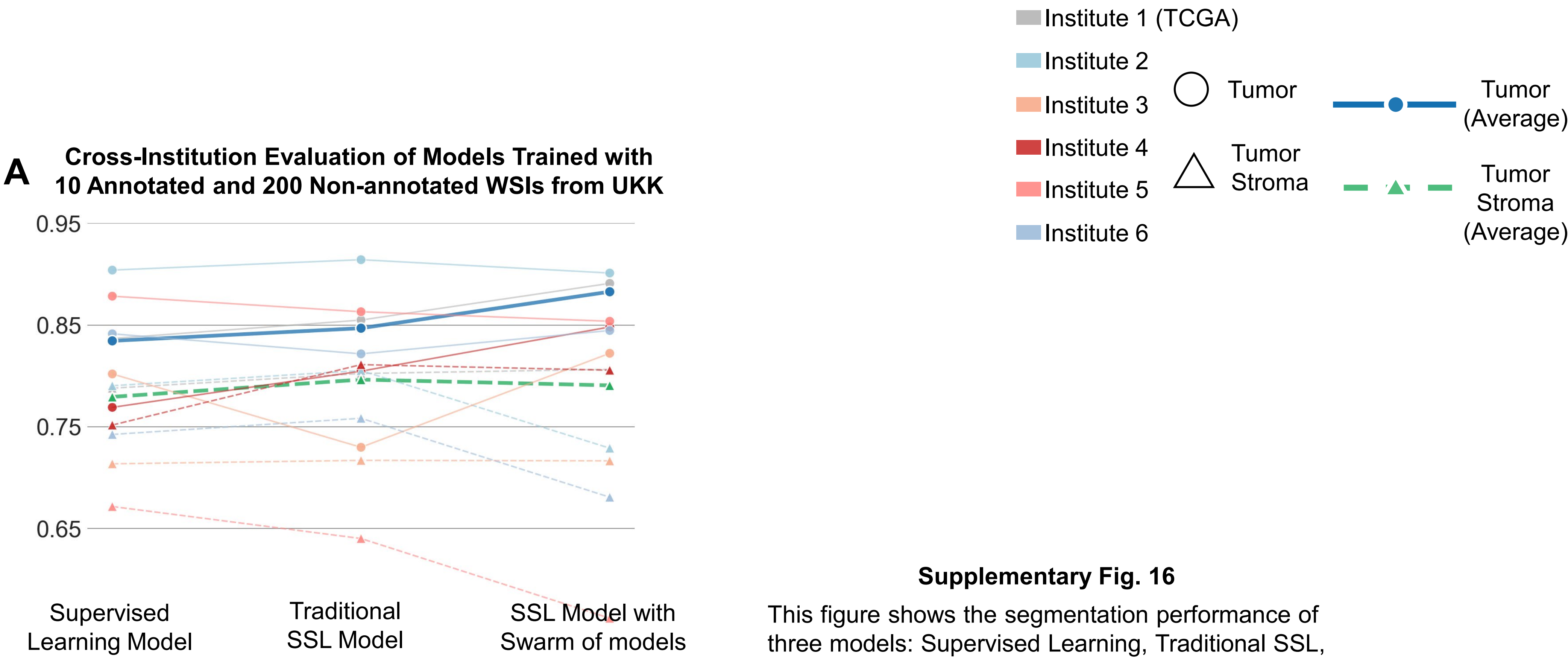

Supplementary Fig. 16

This figure shows the segmentation performance of three models: Supervised Learning, Traditional SSL, and SSL with Swarm Models on tumor and tumor stroma across test datasets from CRAG dataset and four independent institutes. All models were trained using the UKK dataset. The comparison highlights the generalizability and robustness of each model when applied to external, multi-institutional data.

A Similarity Analysis Between Annotated(n=5) and Non-Annotated(n=200) Cases From TCGA (Exp. 1)

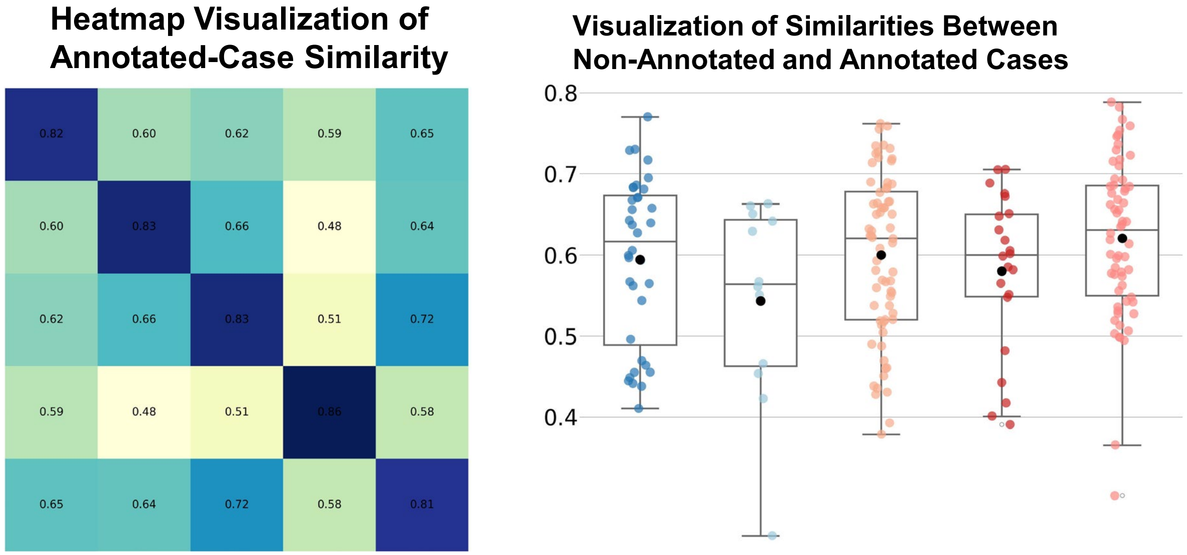

B Similarity Analysis Between Annotated(n=5) and Non-Annotated(n=200) Cases From TCGA (Exp. 2)

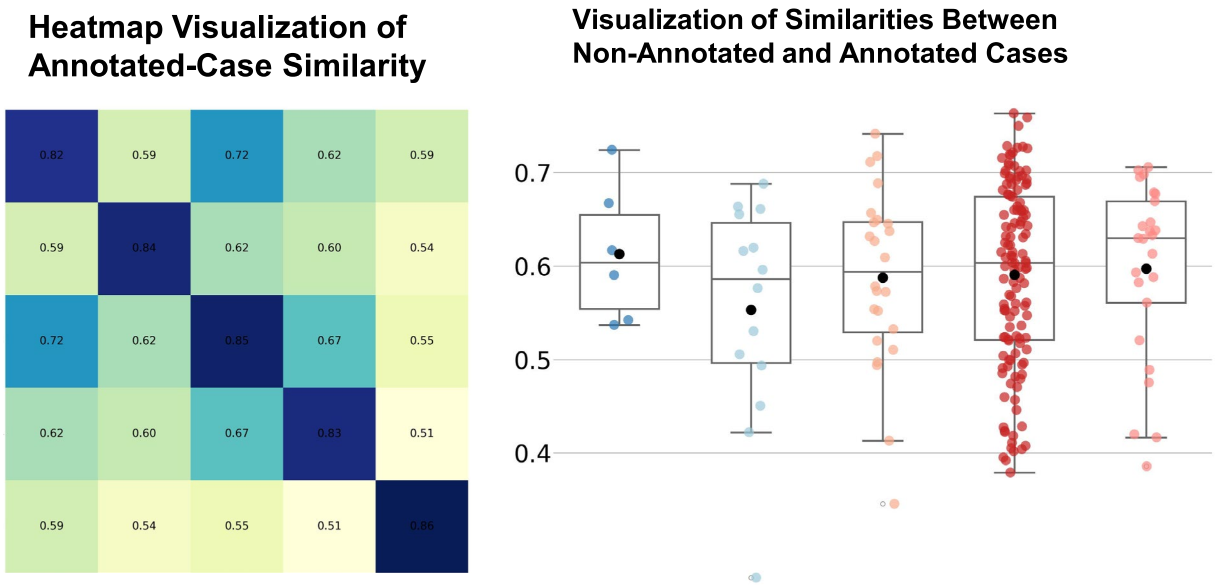

Supplementary Fig. 17 This figure presents the similarity analysis using a dataset setup of 5 annotated cases and 200 non-annotated cases from TCGA, with two different selections of 5 annotated cases. Two heatmaps visualize the similarity between annotated cases in each selection. Two boxplots illustrate the similarity scores between annotated and non-annotated cases, showing which non-annotated cases are most similar to specific annotated cases in each selection.

A Similarity Analysis Between Annotated(n=15) and Non-Annotated(n=200) Cases From TCGA (Exp. 1)

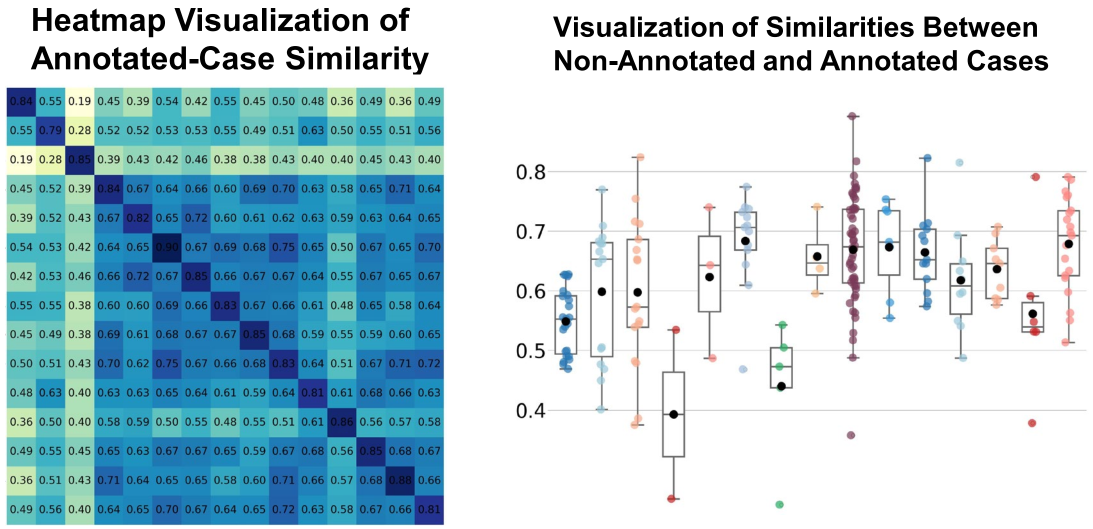

B Similarity Analysis Between Annotated(n=15) and Non-Annotated(n=200) Cases From TCGA (Exp. 2)

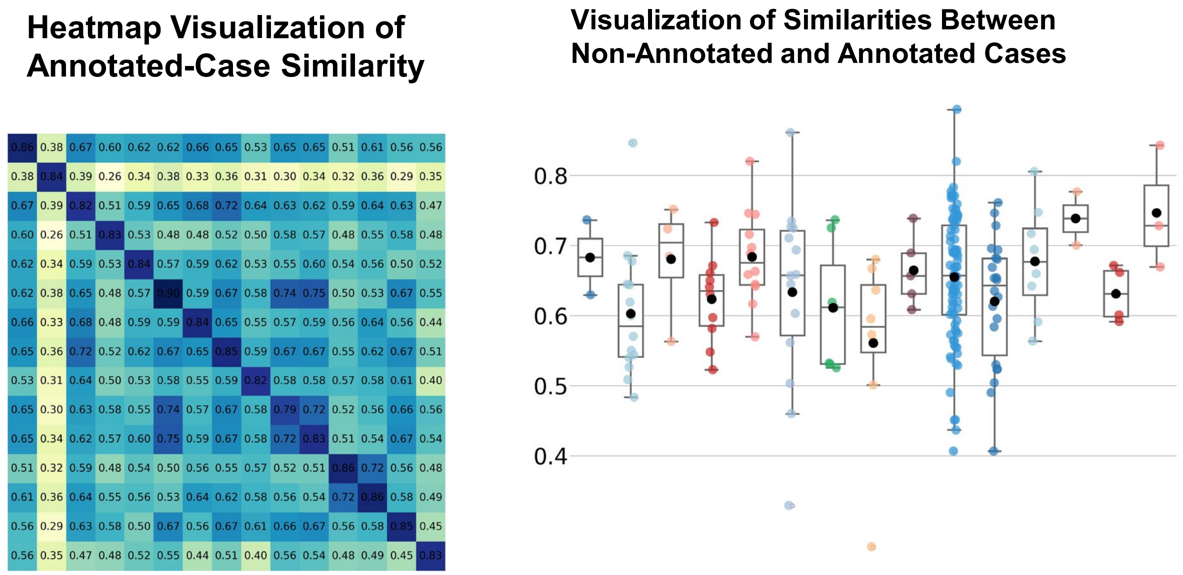

Supplementary Fig. 18 This figure presents the similarity analysis using a dataset setup of 15 annotated cases and 200 non-annotated cases from TCGA, with two different selections of 15 annotated cases. Two heatmaps visualize the similarity between annotated cases in each selection. Additionally, two boxplots illustrate the similarity scores between annotated and non-annotated cases, showing which non-annotated cases are most similar to specific annotated cases in each selection.

Dice Score Performance of the Dataset: Annotated(n=5) and Non-Annotated(n=200) Cases From TCGA

Exp. 1

Model Type	Tumor	Tumor Stroma	Benign Mucosa	Submuc	MUSC PROP / MUC	Vessel	Lymph	Necrosis	Blood	Mucin	Back Ground	Average
Tumor Detection	0.863	0.832									0.871	0.855
Supervised Learning	0.814	0.594	0.863	0.573	0.750	0.613	0.899	0.558	0.638	0.774	0.709	0.708
Traditional SSL	0.784	0.583	0.889	0.670	0.690	0.604	0.930	0.640	0.717	0.743	0.634	0.717
Swarm Method	0.833	0.709	0.886	0.695	0.711	0.559	0.930	0.725	0.756	0.779	0.667	0.750

Exp. 2

Model Type	Tumor	Tumor Stroma	Benign Mucosa	Submuc	MUSC PROP / MUC	Vessel	Lymph	Necrosis	Blood	Mucin	Back Ground	Average
Tumor Detection	0.857	0.839									0.878	0.858
Supervised Learning	0.827	0.586	0.884	0.676	0.783	0.631	0.902	0.756	0.628	0.693	0.793	0.742
Traditional SSL	0.807	0.613	0.882	0.704	0.807	0.546	0.923	0.779	0.756	0.745	0.750	0.756
Swarm Method	0.785	0.709	0.879	0.751	0.786	0.618	0.919	0.761	0.782	0.705	0.774	0.770

Supplementary Fig. 19 This figure presents three tables comparing the Dice scores of different models—Tumor Detection Model, Supervised Learning Model, Traditional SSL Model, and SSL Model with Swarm Models—under a dataset setup of 5 annotated cases and 200 non-annotated cases from TCGA. Each table corresponds to a different selection of 5 annotated cases

Dice Score Performance of the Dataset: Annotated(n=15) and Non-Annotated(n=200) Cases From TCGA

Exp. 1

Model Type	Tumor	Tumor Stroma	Benign Mucosa	Submuc	MUSC PROP / MUC	Vessel	Lymph	Necrosis	Blood	Mucin	Back Ground	Average
Tumor Detection	0.842	0.846									0.855	0.848
Supervised Learning	0.831	0.724	0.909	0.736	0.834	0.848	0.859	0.790	0.782	0.783	0.839	0.813
Traditional SSL	0.807	0.724	0.908	0.785	0.861	0.868	0.918	0.802	0.821	0.804	0.856	0.832
Swarm Method	0.848	0.777	0.901	0.802	0.844	0.864	0.903	0.827	0.824	0.811	0.854	0.841

Exp. 2

Model Type	Tumor	Tumor Stroma	Benign Mucosa	Submuc	MUSC PROP / MUC	Vessel	Lymph	Necrosis	Blood	Mucin	Back Ground	Average
Tumor Detection	0.856	0.853									0.879	0.863
Supervised Learning	0.790	0.655	0.868	0.721	0.707	0.784	0.795	0.848	0.618	0.738	0.810	0.758
Traditional SSL	0.793	0.633	0.846	0.787	0.844	0.801	0.881	0.864	0.736	0.788	0.820	0.799
Swarm Method	0.769	0.691	0.757	0.606	0.741	0.776	0.890	0.873	0.754	0.747	0.825	0.766

Supplementary Fig. 20 This figure presents three tables comparing the Dice scores of different models—Tumor Detection Model, Supervised Learning Model, Traditional SSL Model, and SSL Model with Swarm Models—under a dataset setup of 15 annotated cases and 200 non-annotated cases from TCGA. Each table corresponds to a different selection of 15 annotated cases

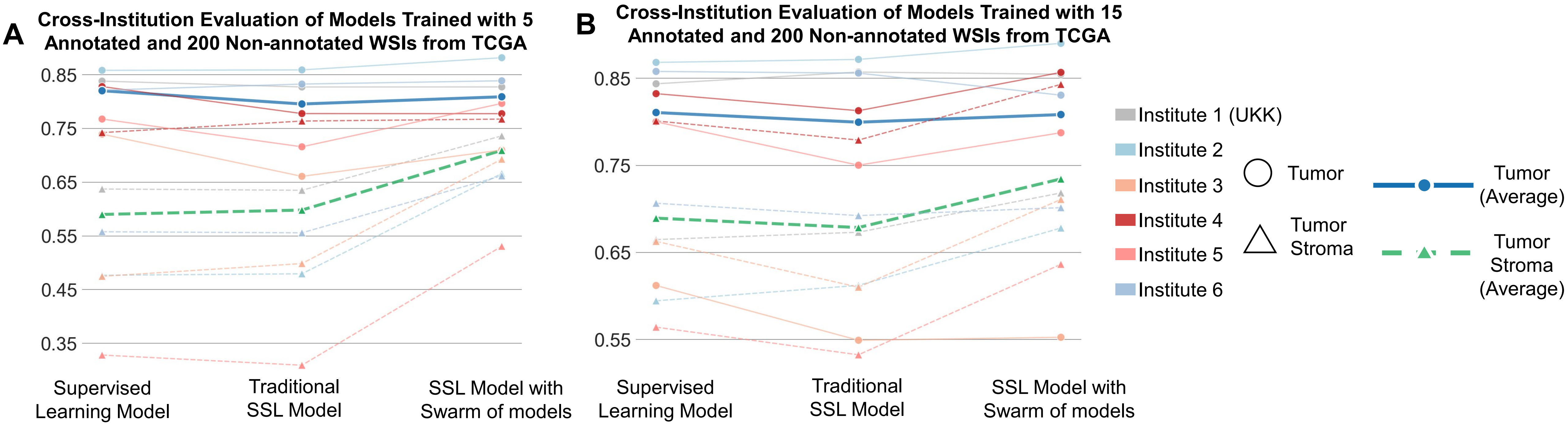

Supplementary Fig. 21

This figure shows the segmentation performance of three models: Supervised Learning, Traditional SSL, and SSL with Swarm Models on tumor and tumor stroma across test datasets from CRAG dataset and four independent institutes. All models were trained using the TCGA dataset. The comparison highlights the generalizability and robustness of each model when applied to external, multi-institutional data.

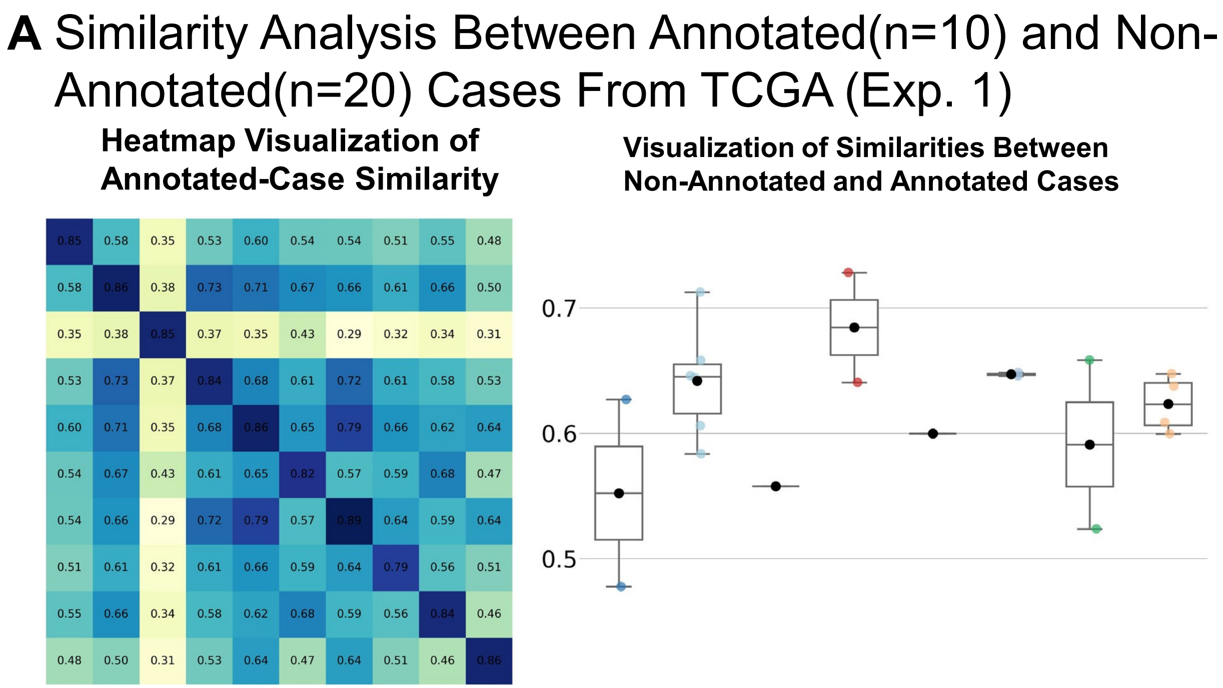

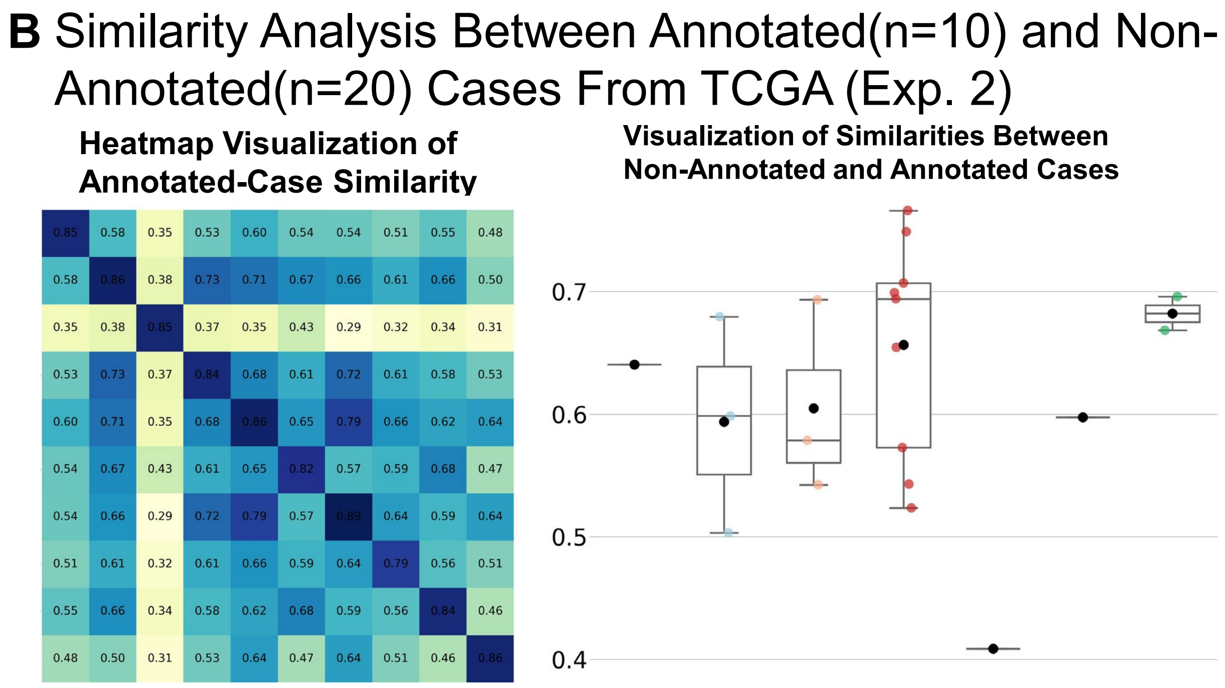

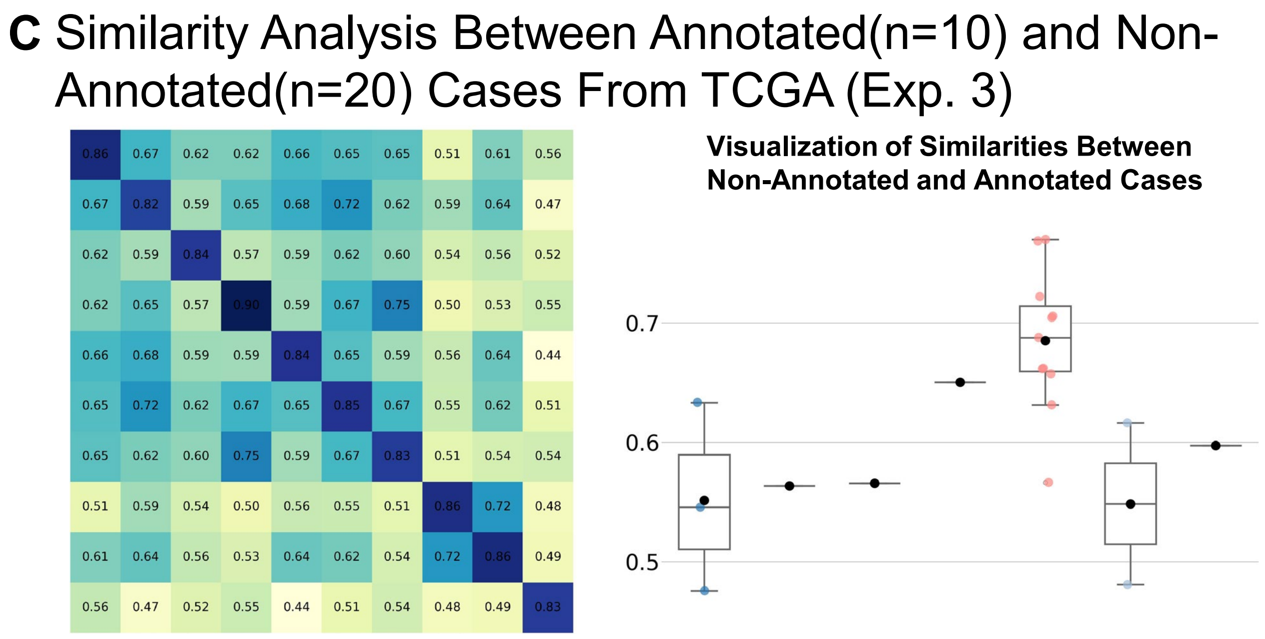

Supplementary Fig. 22 This figure presents the similarity analysis using a dataset setup of 10 annotated cases and 20 non-annotated cases from TCGA, with three different selections of 10 annotated cases. Three heatmaps visualize the similarity between annotated cases in each selection. Three boxplots illustrate the similarity scores between annotated and non-annotated cases, showing which non-annotated cases are most similar to specific annotated cases in each selection.

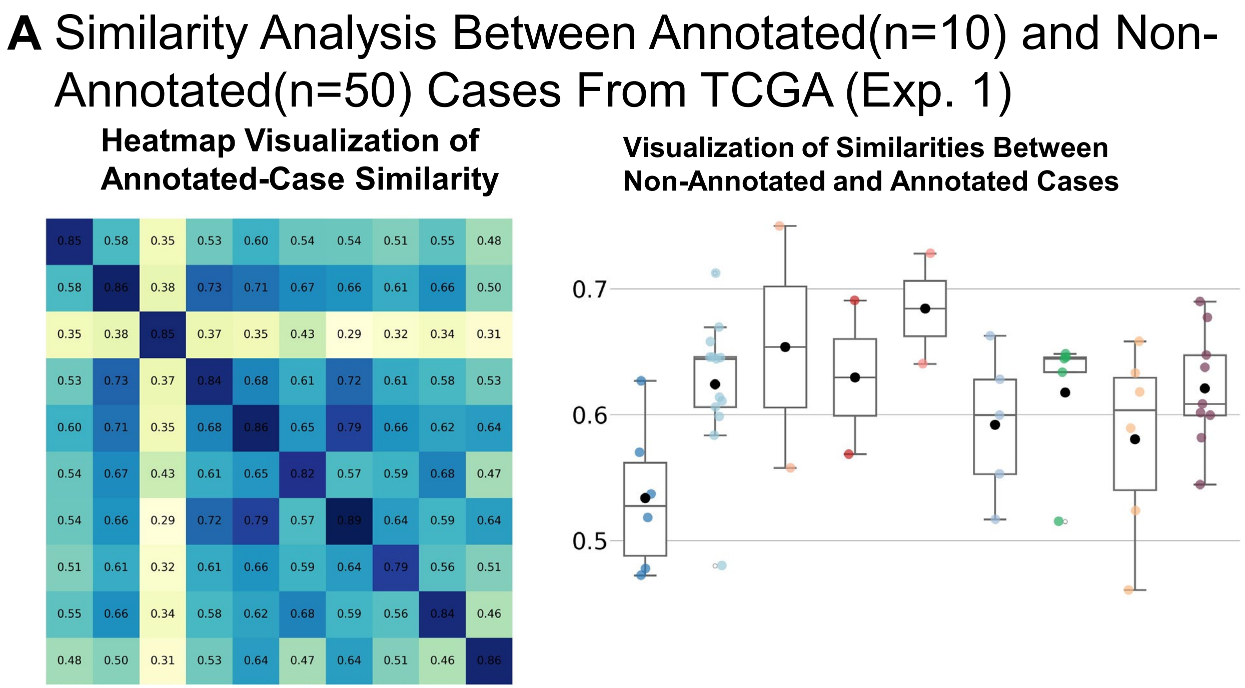

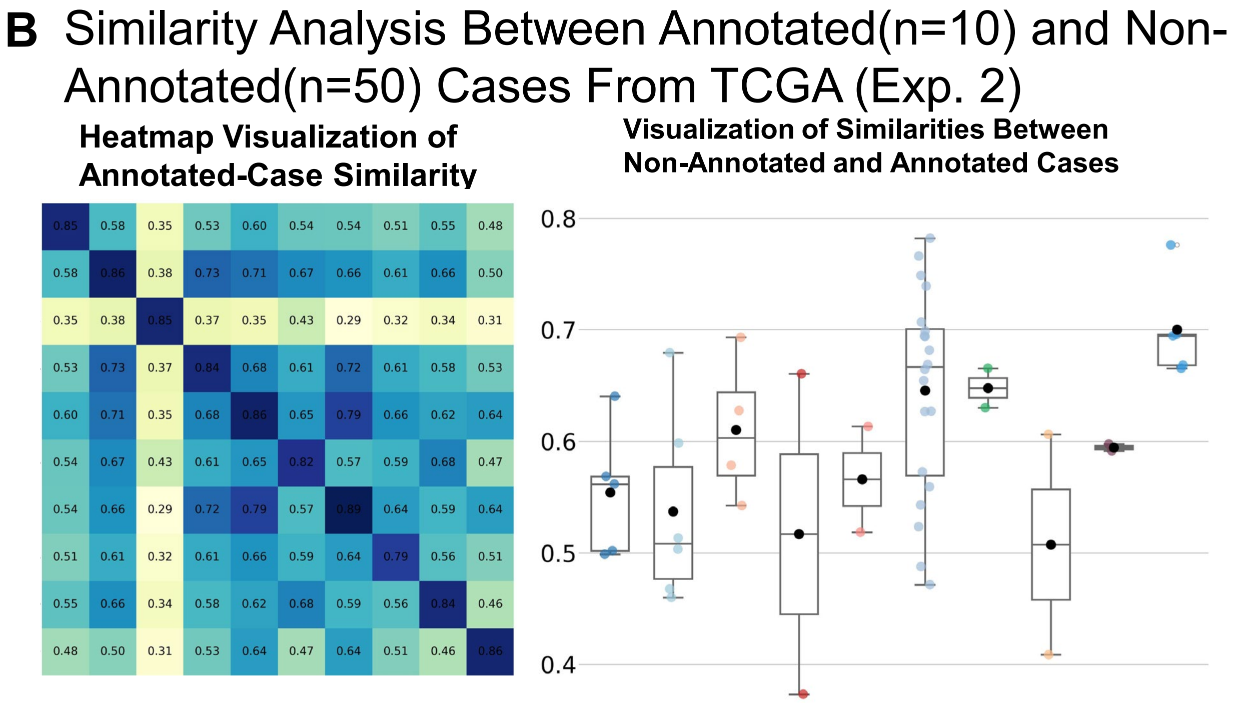

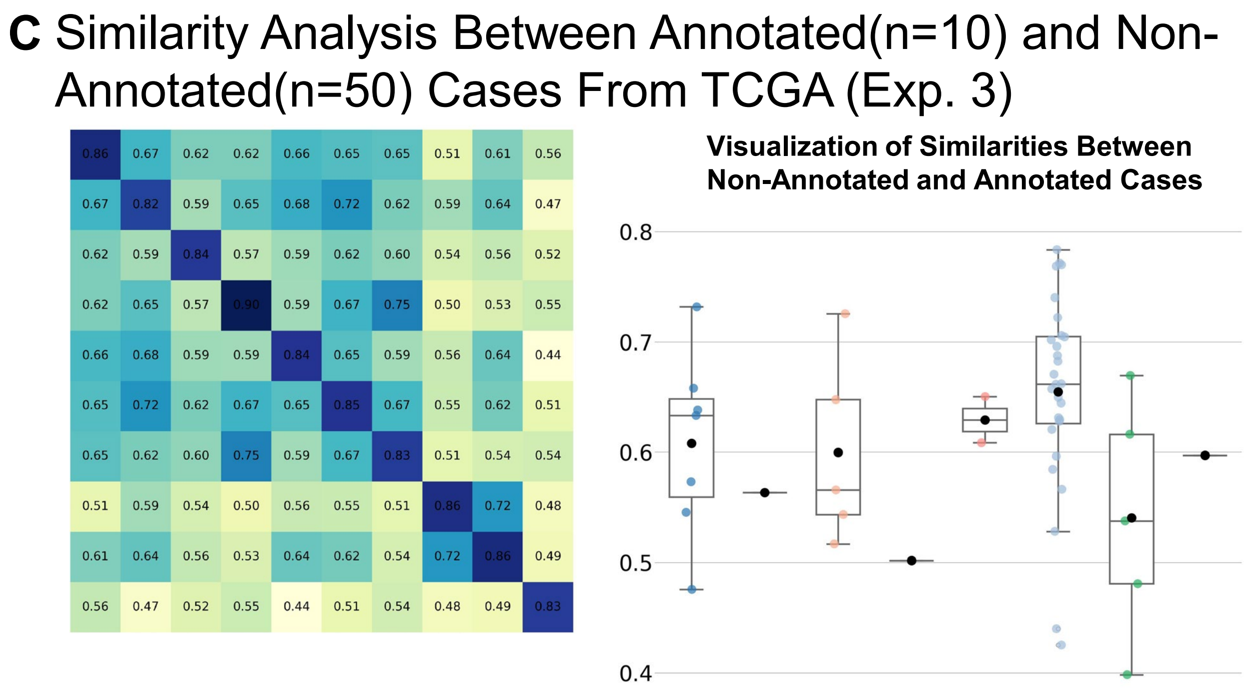

Supplementary Fig. 23 This figure presents the similarity analysis using a dataset setup of 10 annotated cases and 50 non-annotated cases from TCGA, with three different selections of 10 annotated cases. Three heatmaps visualize the similarity between annotated cases in each selection. Three boxplots illustrate the similarity scores between annotated and non-annotated cases, showing which non-annotated cases are most similar to specific annotated cases in each selection.

A Similarity Analysis Between Annotated(n=10) and Non-Annotated(n=100) Cases From TCGA (Exp. 1)

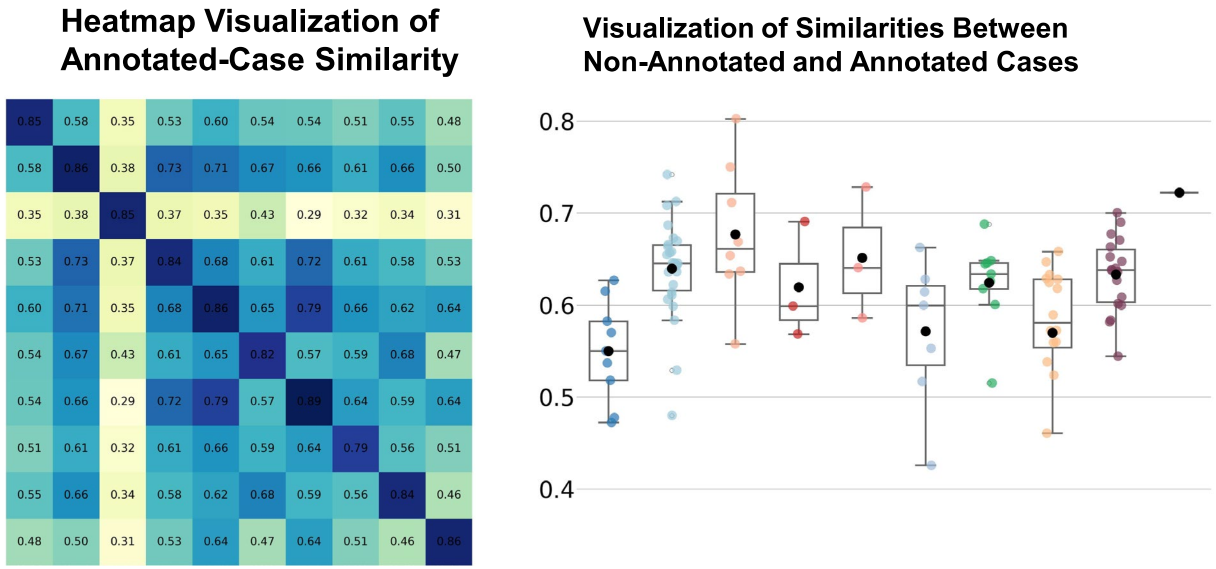

B Similarity Analysis Between Annotated(n=10) and Non-Annotated(n=100) Cases From TCGA (Exp. 2)

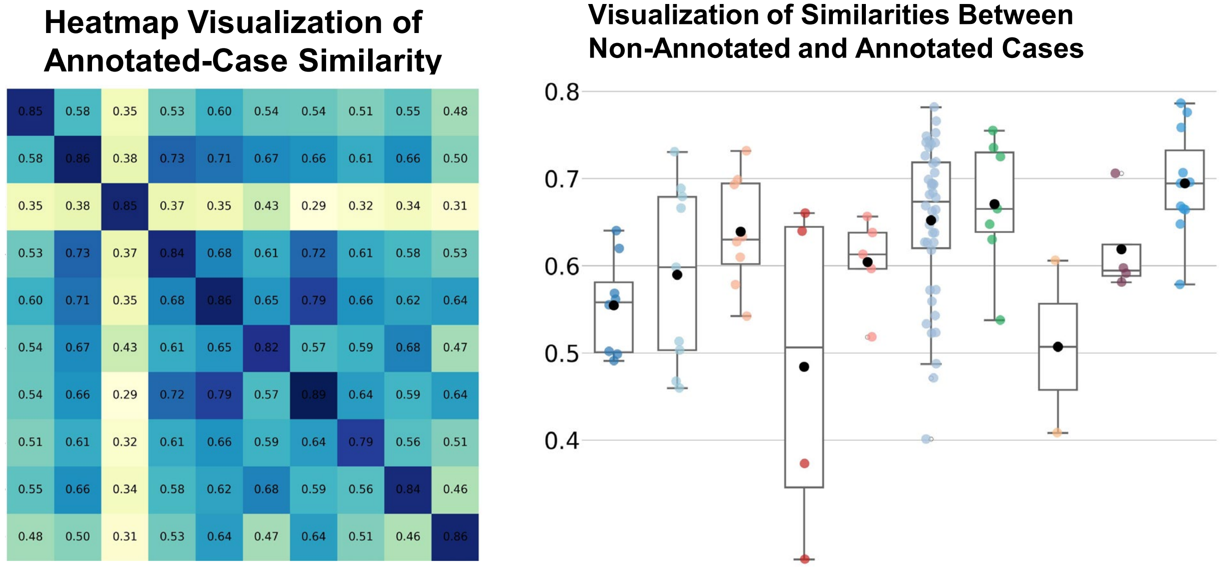

C Similarity Analysis Between Annotated(n=10) and Non-Annotated(n=100) Cases From TCGA (Exp. 3)

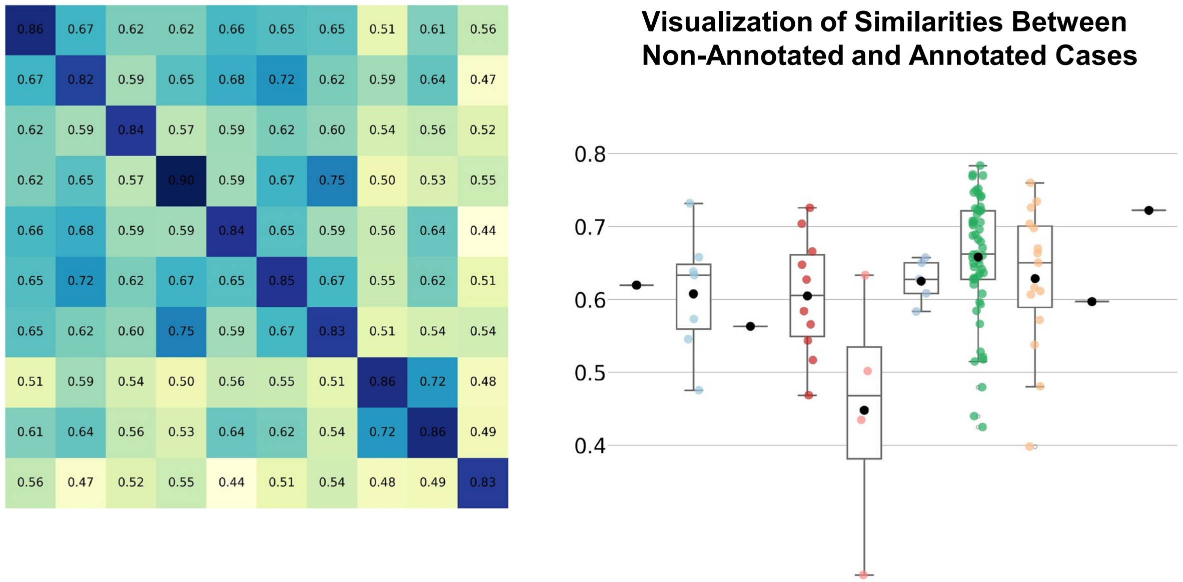

Supplementary Fig. 24 This figure presents the similarity analysis using a dataset setup of 10 annotated cases and 100 non-annotated cases from TCGA, with three different selections of 10 annotated cases. Three heatmaps visualize the similarity between annotated cases in each selection. Three boxplots illustrate the similarity scores between annotated and non-annotated cases, showing which non-annotated cases are most similar to specific annotated cases in each selection.

Dice Score Performance of the Dataset: Annotated(n=10) and Non-Annotated(n=20) Cases From TCGA

Exp. 1

Model Type	Tumor	Tumor Stroma	Benign Mucosa	Submuc	MUSC PROP / MUC	Vessel	Lymph	Necrosis	Blood	Mucin	Back Ground	Average
Tumor Detection	0.858	0.875									0.903	0.879
Supervised Learning	0.755	0.621	0.885	0.727	0.683	0.837	0.906	0.618	0.709	0.664	0.846	0.750
Traditional SSL	0.749	0.669	0.891	0.835	0.845	0.850	0.914	0.613	0.749	0.668	0.863	0.786
Swarm Method	0.786	0.657	0.802	0.732	0.724	0.853	0.911	0.522	0.732	0.763	0.869	0.759

Exp. 2

Model Type	Tumor	Tumor Stroma	Benign Mucosa	Submuc	MUSC PROP / MUC	Vessel	Lymph	Necrosis	Blood	Mucin	Back Ground	Average
Tumor Detection	0.835	0.865									0.884	0.861
Supervised Learning	0.820	0.711	0.895	0.697	0.866	0.745	0	0.752	0.769	0.682	0.780	0.702
Traditional SSL	0.806	0.669	0.909	0.642	0.849	0.740	0	0.669	0.746	0.721	0.751	0.682
Swarm Method	0.786	0.764	0.882	0.594	0.858	0.792	0	0.788	0.741	0.761	0.778	0.704

Exp. 3

Model Type	Tumor	Tumor Stroma	Benign Mucosa	Submuc	MUSC PROP / MUC	Vessel	Lymph	Necrosis	Blood	Mucin	Back Ground	Average
Tumor Detection	0.847	0.867									0.888	0.867
Supervised Learning	0.769	0.666	0.848	0.726	0.750	0.728	0.860	0.846	0.695	0.728	0.765	0.762
Traditional SSL	0.698	0.673	0.906	0.795	0.746	0.772	0.902	0.797	0.651	0.598	0.731	0.752
Swarm Method	0.782	0.660	0.878	0.732	0.849	0.789	0.897	0.859	0.749	0.710	0.787	0.790

Supplementary Fig. 25 This figure presents three tables comparing the Dice scores of different models—Tumor Detection Model, Supervised Learning Model, Traditional SSL Model, and SSL Model with Swarm Models—under a dataset setup of 10 annotated cases and 20 non-annotated cases from TCGA. Each table corresponds to a different selection of 10 annotated cases

Dice Score Performance of the Dataset: Annotated(n=10) and Non-Annotated(n=50) Cases From TCGA

Exp. 1

Model Type	Tumor	Tumor Stroma	Benign Mucosa	Submuc	MUSC PROP / MUC	Vessel	Lymph	Necrosis	Blood	Mucin	Back Ground	Average
Tumor Detection	0.858	0.875									0.903	0.879
Supervised Learning	0.755	0.621	0.885	0.727	0.683	0.837	0.906	0.618	0.709	0.664	0.846	0.750
Traditional SSL	0.748	0.503	0.874	0.830	0.760	0.835	0.932	0.572	0.739	0.756	0.842	0.763
Swarm Method	0.800	0.650	0.868	0.796	0.821	0.867	0.917	0.580	0.756	0.783	0.867	0.790

Exp. 2

Model Type	Tumor	Tumor Stroma	Benign Mucosa	Submuc	MUSC PROP / MUC	Vessel	Lymph	Necrosis	Blood	Mucin	Back Ground	Average
Tumor Detection	0.835	0.865									0.884	0.861
Supervised Learning	0.820	0.711	0.895	0.697	0.866	0.745	0	0.752	0.769	0.682	0.780	0.702
Traditional SSL	0.819	0.752	0.886	0.750	0.887	0.804	0	0.790	0.832	0.756	0.800	0.734
Swarm Method	0.782	0.764	0.897	0.722	0.890	0.772	0	0.791	0.794	0.764	0.768	0.722

Exp. 3

Model Type	Tumor	Tumor Stroma	Benign Mucosa	Submuc	MUSC PROP / MUC	Vessel	Lymph	Necrosis	Blood	Mucin	Back Ground	Average
Tumor Detection	0.847	0.867									0.888	0.867
Supervised Learning	0.769	0.666	0.848	0.726	0.750	0.728	0.860	0.846	0.695	0.728	0.765	0.762
Traditional SSL	0.789	0.707	0.891	0.787	0.816	0.789	0.899	0.841	0.798	0.803	0.768	0.808
Swarm Method	0.808	0.716	0.872	0.830	0.855	0.819	0.874	0.881	0.749	0.747	0.819	0.816

Supplementary Fig. 26 This figure presents three tables comparing the Dice scores of different models—Tumor Detection Model, Supervised Learning Model, Traditional SSL Model, and SSL Model with Swarm Models—under a dataset setup of 10 annotated cases and 50 non-annotated cases from TCGA. Each table corresponds to a different selection of 10 annotated cases

Dice Score Performance of the Dataset: Annotated(n=10) and Non-Annotated(n=100) Cases From TCGA

Exp. 1

Model Type	Tumor	Tumor Stroma	Benign Mucosa	Submuc	MUSC PROP / MUC	Vessel	Lymph	Necrosis	Blood	Mucin	Back Ground	Average
Tumor Detection	0.858					0.875					0.903	0.879
Supervised Learning	0.755	0.621	0.885	0.727	0.683	0.837	0.906	0.618	0.709	0.664	0.846	0.750
Traditional SSL	0.766	0.570	0.902	0.752	0.808	0.826	0.940	0.684	0.797	0.789	0.853	0.790
Swarm Method	0.803	0.648	0.860	0.757	0.745	0.846	0.906	0.610	0.785	0.765	0.875	0.782

Exp. 2

Model Type	Tumor	Tumor Stroma	Benign Mucosa	Submuc	MUSC PROP / MUC	Vessel	Lymph	Necrosis	Blood	Mucin	Back Ground	Average
Tumor Detection	0.835					0.865					0.884	0.861
Supervised Learning	0.820	0.711	0.895	0.697	0.866	0.745	0	0.752	0.769	0.682	0.780	0.702
Traditional SSL	0.755	0.733	0.896	0.733	0.886	0.808	0	0.741	0.834	0.771	0.804	0.724
Swarm Method	0.797	0.749	0.897	0.733	0.886	0.775	0	0.782	0.836	0.764	0.746	0.724

Exp. 3

Model Type	Tumor	Tumor Stroma	Benign Mucosa	Submuc	MUSC PROP / MUC	Vessel	Lymph	Necrosis	Blood	Mucin	Back Ground	Average
Tumor Detection	0.847					0.867					0.888	0.867
Supervised Learning	0.769	0.666	0.848	0.726	0.750	0.728	0.860	0.846	0.695	0.728	0.765	0.762
Traditional SSL	0.775	0.674	0.889	0.786	0.807	0.809	0.884	0.869	0.798	0.788	0.791	0.806
Swarm Method	0.768	0.686	0.894	0.772	0.830	0.807	0.888	0.873	0.787	0.777	0.814	0.809

Supplementary Fig. 27 This figure presents three tables comparing the Dice scores of different models—Tumor Detection Model, Supervised Learning Model, Traditional SSL Model, and SSL Model with Swarm Models—under a dataset setup of 10 annotated cases and 100 non-annotated cases from TCGA. Each table corresponds to a different selection of 10 annotated cases

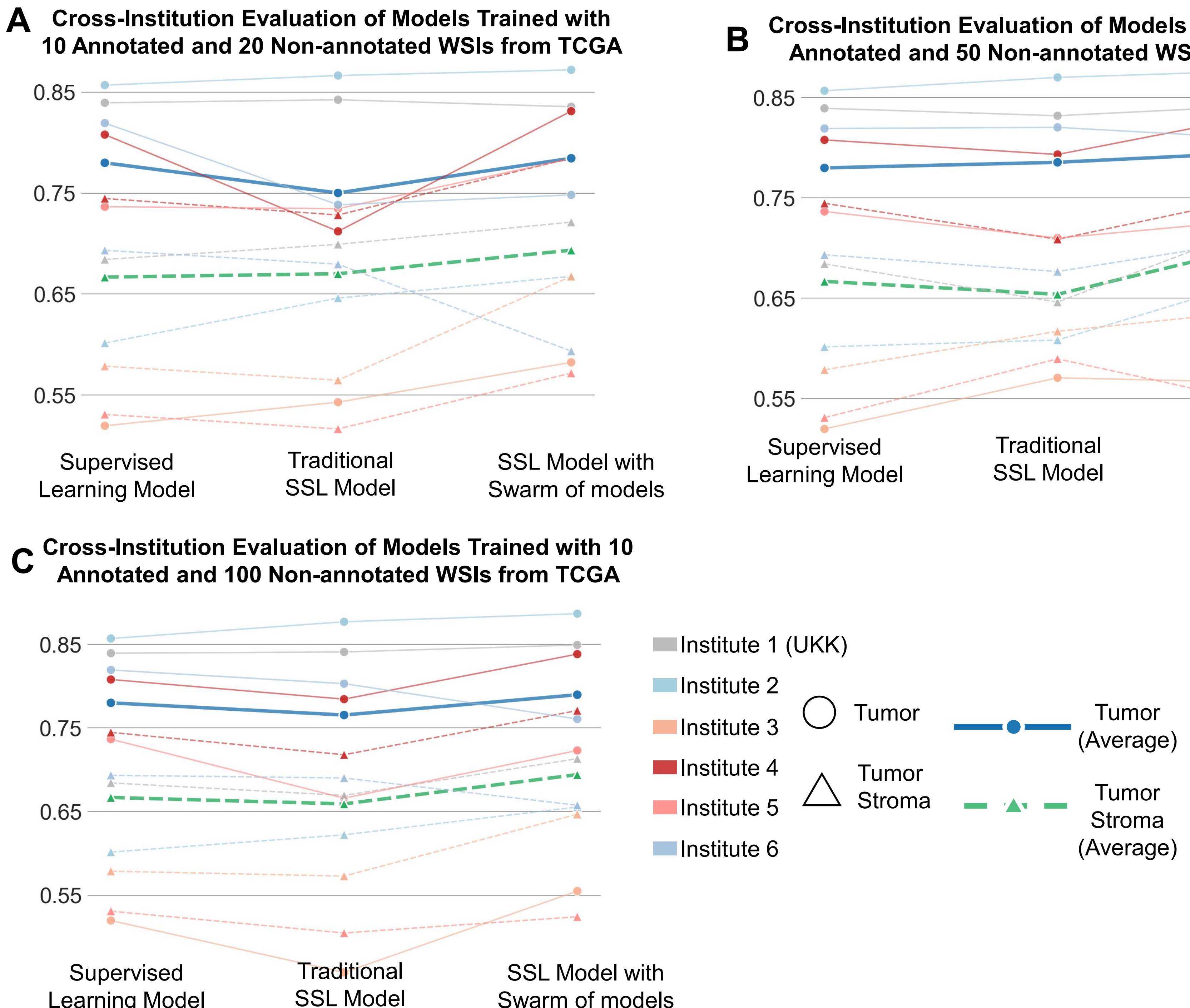

Supplementary Fig. 28

This figure shows the segmentation performance of three models: Supervised Learning, Traditional SSL, and SSL with Swarm Models on tumor and tumor stroma across test datasets from CRAG dataset and four independent institutes. All models were trained using the TCGA dataset. The comparison highlights the generalizability and robustness of each model when applied to external, multi-institutional data.

Training Time Comparison (in GPU Hours) across Different Methods and Models (Exp. 1)

Method	Model	GPU hour / Epoch	Epoch number	GPU hours
S-o-M Based SSL	Supervised Model	0.089	36	3.204
	Tumor Detection Model	0.142	36	5.112
	Single Case Models (10 Cases)	0.038	36	1.368
	SSL Model	0.883	36	31.788
	**In Total**			**41.472**
Traditional Pseudo-Label SSL	Supervised Model	0.089	36	3.204
	SSL Model	0.883	36	31.788
	**In Total**			**34.992**
Supervised Learning	Supervised Model	0.089	36	3.204
	**In Total**			**3.204**

Supplementary Fig. 29 This table summarizes the training time (in GPU hours) for each model and the total time required by four different methods. All experiments were conducted on NVIDIA A100 GPUs with 80 GB memory.
